# Lineage Diversification and Population Dynamics of the Qinghai Toad-Headed Agama (*Phrynocephalus vlangalii*) on the Qinghai–Tibet Plateau, with Particular Attention to the Northern Slope of the Kunlun–Arjin Mountains

**DOI:** 10.3390/ani15030400

**Published:** 2025-01-31

**Authors:** Rui Xu, Qi Song, Dali Chen, Xianguang Guo

**Affiliations:** 1 Chengdu Institute of Biology, Chinese Academy of Sciences, Chengdu 610213, China; xurui@cib.ac.cn; 2 Chengdu Library and Information Center, Chinese Academy of Sciences, Chengdu 610299, China; songq@clas.ac.cn; 3 Department of Pathogenic Biology, West China School of Basic Medical Sciences and Forensic Medicine, Sichuan University, Chengdu 610041, China; chendali@scu.edu.cn

**Keywords:** *Phrynocephalus*, genetic diversity, morphological characters, ecological niche modeling, cryptic diversity, phylogeography

## Abstract

The Kunlun, Arjin, and Qilian mountain ranges mark the northern edge of the Qinghai–Tibet Plateau (QTP), where rapid uplift and Quaternary glacial cycles have shaped a unique cold desert ecosystem and species distribution. Despite sampling challenges, phylogeographic studies are crucial for understanding reptile populations such as the Qinghai toad-headed agama (*Phrynocephalus vlangalii*), a viviparous lizard with limited dispersal and multiple subspecies in the northeastern QTP. Our fieldwork identified populations of *P. vlangalii* on the northern slope of the Kunlun–Arjin Mountains, similar to the controversial subspecies *P. v. lidskii*. We analyzed 130 individuals from the northern slope of the Kunlun–Arjin–Qilian Mountains and 253 individuals from GenBank, using three mitochondrial genes and two nuclear genes to assess intraspecific differentiation and demographic history. We found high haplotype diversity and low nucleotide diversity in *P. vlangalii*, with phylogenetic analyses revealing six distinct clades. Clade VI, confirmed as *P. v. lidskii*, and Clade IV, a new genetic lineage, were identified alongside three recognized subspecies. Genetic variation was largely attributed to clade splitting, indicating significant divergence. The Mantel test indicated that geographical and environmental factors drove population differentiation. Bayesian molecular clock analysis suggested that the most recent common ancestor of *P. vlangalii* lived 2.55 million years ago, influenced by the Qinghai–Tibet Movement and glacial cycles. Demographic history and ecological niche modeling (ENM) indicated no population decline during the Last Glacial Maximum, supporting the glacial maximum expansion model, with ENM predicting future habitat expansion for *P. vlangalii*. In addition, morphological data from 13 meristic and 15 metric characters confirmed clade differences. Our findings significantly advance our understanding of *P. vlangalii* diversification, population dynamics and response to geological and climatic changes in the QTP.

## Simple Summary

This study investigates the phylogeography and population genetics of the Qinghai toad-headed agama (*Phrynocephalus vlangalii*) on the Qinghai–Tibet Plateau. We analyzed genetic data from 130 new individuals and from 253 individuals from GenBank and identified six distinct clades, including a new genetic lineage and the subspecies *P. v. lidskii* on the northern slope of the Kunlun–Arjin Mountains. The study found high genetic diversity and significant divergence between the clades, driven by geographical and environmental variables. The ancestry of the species dates back 2.55 million years, influenced by geological movements and glacial cycles. There was no population decline during the Last Glacial Maximum, and ecological niche modeling predicts future habitat expansion. Morphological data also confirmed clade differences, improving our understanding of *P. vlangalii* diversification and adaptation to geological and climatic changes in the region.

## 1. Introduction

Patterns of population diversity and geographical distribution in the contemporary world are influenced by historical geological events and climate fluctuations, either in isolation or in combination [1,2,3,4]. Since the Quaternary period (2.6 million years ago, Ma), the cycles of glacial and interglacial periods have caused global climate fluctuations, while the Qinghai–Tibet Plateau (QTP) has undergone rapid uplift in three phases: the Qinghai–Tibet Movement, the Kunhuang Movement, and the Gonghe Movement [5]. These events have profoundly affected the geological changes around the plateau and the differentiation and distribution patterns of its species [6].

Mountains are known for their unique biodiversity. The Kunlun–Arjin–Qilian mountain range marks the boundary between the arid and semi-arid regions and the QTP. The region’s high-altitude deserts, grasslands, glaciers, and snow-capped mountains support a unique ecosystem and are home to rare alpine species such as the Tibetan antelope (*Pantholops hodgsonii)*, the Tibetan wild ass (*Equus kiang*), the Tibetan gazelle (*Procapra picticaudata*), and the black-necked crane (*Grus nigricollis*) [7,8,9]. However, due to its remoteness, poor transport, and collection challenges, this area has long received minimal scientific attention, with recent studies focusing on flora, birds, and mammals, leaving amphibians and reptiles largely unstudied.

The viviparous species of the genus *Phrynocephalus* are a remarkable group of reptiles on the QTP, including *P*. *vlangalii*, *P. theobaldi*, *P. erythrurus*, *P. putjatai* and *P. forsythii* [10]. These plateau lizards form a monophyletic group on the phylogenetic tree, with their species differentiation and geographical distribution primarily influenced by the geological movements of the QTP [11,12]. Ongoing scientific research is refining our understanding of the biodiversity of the QTP (e.g., [12,13,14,15,16,17]).

*Phrynocephalus vlangalii*, first described by Strauch in 1876 from the vicinity of Qinghai Lake [10], is well adapted to the cold, high altitude and dry sandy environments of the QTP. This species occurs at altitudes ranging from 2300 to 4500 m (our field observation), making it an excellent subject for investigating geographical patterns in temperature-variable faunal systems on the QTP and its adjacent regions. Building on Strauch’s work, Bedriaga [18] later described two new species and six varieties of *P*. *vlangalii* based on morphological characters from areas around Qinghai Lake. These include *P. reldoe* from the southern part of Qinghai Lake, *P. roborowskii* from the Qaidam Basin, *P*. *v.* var. *nanschanica* from the Nanshan Mountains, *P. v.* var. *grombtschewskii* from the salty Gobi Desert in the southern part of Qinghai Lake, *P*. *v*. var. *geckoides* from the Tschumar or Qumar River, *P. v.* var. *parva* from Moron Township and the Tuotuo River, *P*. *v*. var. *pylzowi*, and *P*. *v*. var. *lidskii* from the Yutian–Keria Mountains. Subsequently, Jiang et al. [19] identified a new subspecies *P. v. hongyuanensis*, based on scale differences. However, the morphological classification system has been continuously challenged and remains controversial [20,21,22,23,24,25,26]. Pope [20] classified *Phrynocephalus* from different regions of Qinghai as *P. vlangalii*, based on the position of their external nostrils and abdominal pigmentation, and suggested that it may be better not to distinguish between subspecies of *P. vlangalii*. Kentang Zhao [21,22,23] largely agreed with Pope’s view. Zhao and Adler [24] pointed out that it remains to be confirmed whether *P. reldoe* and *P. roborowskii* are valid species. Peters [25] briefly discussed the classification issue of *P. vlangalii* and largely supported Zarewsky’s view.

Jin et al. [27] pioneered the use of mitochondrial DNA (mtDNA) genetic markers to clarify the phylogenetic relationships of the viviparous group within the genus *Phrynocephalus* on the QTP. Their research recognized three distinct subspecies of *P. vlangalii* with discontinuous distribution and evidence of long-term genetic isolation: *P*. *v. vlangalii*, *P. v. nanschanica*, and *P*. *v. pylzowi*. This finding questioned the validity of other proposed subspecies and suggested reclassifications, including *P. v. lidskii* as a synonym of *P. forsythii, P. v. hongyuanensis* as a synonym of *P. v. pylzowi, P. v. grombtschewskii* and *P. roborowskii* as synonyms of *P. v. vlangalii*, and the establishment of *P. v. parva* as a subspecies of *Phrynocephalus erythrurus* (*P. erythrurus parva*) [27,28]. More recently, Chen et al. [12] discovered a previously undescribed and genetically distinct population in Gonghe County, Qinghai. However, their study did not extend to the northern slopes of the Kunlun and Arjin mountains, where unique *Phrynocephalus* specimens were collected. These lizards show clear morphological differences from sympatric *P. forsythii* and a greater similarity to *P*. *vlangalii*. This challenges their previous classification as *P. v. lidskii* and adds to the ongoing debate about their taxonomic status.

In this study, we sampled *P*. *vlangalii* from the northern slope of the Kunlun–Arjin–Qilian Mountains (NKAQ) and assessed intraspecific diversity using molecular data (mtDNA and nuDNA), morphological data, and environmental data. Our objectives were as follows: (i) to investigate the genetic structure and geographical distribution of *P*. *vlangalii* populations; (ii) to compare morphological differences among populations; (iii) to assess the validity of *P. v. lidskii*; and (iv) to explore the evolutionary history and population dynamics in response to environmental climate change.

## 2. Materials and Methods

### 2.1. Sample Collection and DNA Sequencing Procedures

**Figure 1:**
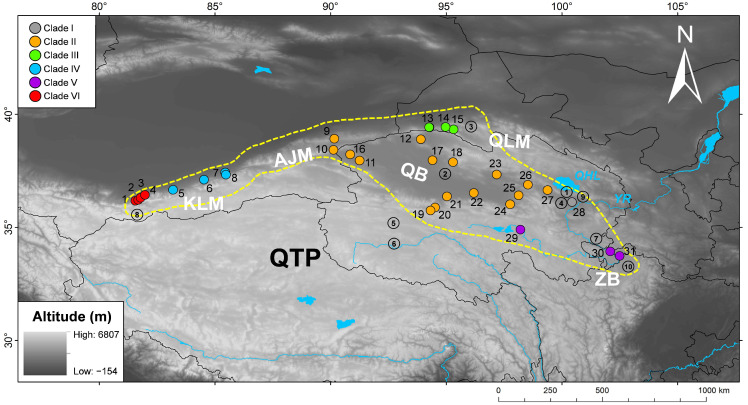
Collection sites for *P. vlangalii* samples used in this study. The sites are numbered in accordance with Table 1 and Table S1, with clades distinguished by different colors, as referenced in Figure 2. Sites 16–31 were sourced from previous research by Chen et al. [12]. The following abbreviations represent the geographical features associated with the collection sites: KLM, Kunlun Mountains; AJM, Arjin Mountains; QLM, Qilian Mountains; QTP, Qinghai–Tibet Plateau; QB, Qaidam Basin; ZB, Zoige Basin; YR, Yellow River; QHL, Qinghai Lake. The yellow dashed line represents the approximate distribution area of *P. vlangalii*. Numbers in circle correspond to exact (solid line) and approximate (dashed line) location of the type localities of taxa described within *P. vlangalii* in the past, as follows: 1—*vlangalii* Strauch, 1876—Qinghai, Qinghai Lake [Kuku-noor]; 2—*roborowskii* Bedriaga, 1906—Qinghai, Qaidam Basin [Provinz Zaidam]; 3—*nanschanica* Bedriaga, 1906—Nanshan mountains [Nan-shan-Gebirge]; 4—*grombtschewskii* Bedriaga 1909—Qinghai, Qaidam Basin, Salinized Gobi Desert [Dabsun-Gobi]; 5 *geckoides* Bedriaga 1909—Qinghai, probably Tschumar, or Qumar River [Estuary of the Tschurmyn River]; 6—*parva* Bedriaga 1909—Qinghai, Moron Us and Tuotuo rivers [Dytschu]; 7—*pylzowi* Bedriaga, 1909—Qinghai, the junction of Qinghai, Gansu, and Sichuan provinces [Hoang-ho-Quellgebiet]; 8—*lidskii* Bedriaga, 1909—southern Xinjiang Uygur Autonomous Region, northern Xizang (Tibet) Autonomous Region, Yutian Mountains [Keria-Gebirge]; 9—*reldoe* Bedriaga, 1909—Qinghai, Balekun Gomi or Kyikug, near Gong He County (36°20′ N, 100°60′ E), south of Qinghai (Kuku-Nor) Lake [Gomi]; 10—*hongyuanensis* Zhao, 1980—Sichuan, Hongyuan County (33°03′N, 102°37′E).

This study is based on data from a total of 383 individuals of *P. vlangalii* collected from 31 localities. Of these, 130 samples were newly collected from 15 sites in the NKAQ (Figure 1), while the remaining 253 were obtained from the previous study by Chen et al. [12]. In accordance with the animal use protocol approved by the Chengdu Institute of Biology (CIB) of the Chinese Academy of Sciences, two to three lizards from each site were euthanized via intraperitoneal injection of an overdose of pentobarbital sodium, and voucher specimens were preserved for reference. All other lizards were released immediately after measurement and tail end clipping at the capture site. Liver tissue was also collected from euthanized individuals and preserved in 95% ethanol and stored at −20 °C. Voucher specimens were deposited at the CIB. Full details of samples can be found in Supplementary Materials Tables S1 and S2.

**Table 1:** Location information for newly collected *P. vlangalii* samples in this study.

Site Number	n	Locality	Clade	Latitude	Longitude	Altitude (m)
1	9	Yutian County, Xinjiang	VI	36.18	81.56	2575
2	3	Yutian County, Xinjiang	VI	36.22	81.68	2835
3	12	Yutian County, Xinjiang	VI	36.31	81.78	3088
4	5	Yutian County, Xinjiang	VI	36.44	81.97	2629
5	8	Minfeng County, Xinjiang	IV	36.67	83.18	2584
6	11	Minfeng County, Xinjiang	IV	37.11	84.53	2391
7	6	Qiemo County, Xinjiang	IV	37.43	85.44	2343
8	12	Qiemo County, Xinjiang	IV	37.33	85.47	2767
9	6	Ruoqiang County, Xinjiang	II	38.43	90.12	3053
10	4	Ruoqiang County, Xinjiang	II	38.92	90.16	3226
11	5	Huatugou Town, Qinghai	II	37.96	91.25	3008
12	14	Sugan Lake, Gansu	II	38.90	93.90	2800
13	14	Akesai County, Gansu	III	39.43	94.26	2539
14	9	Subei County, Gansu	III	39.34	95.32	3068
15	12	Subei County, Gansu	III	39.44	94.97	2489

For comparison with the data of Chen et al. [12], three mitochondrial DNA (mtDNA) gene fragments were amplified and sequenced: 16S ribosomal RNA (16S rRNA, 480 bp), NADH dehydrogenase subunit 2 (ND2) together with an adjacent fragment of the complete tRNA^Trp^ and partial tRNA^Ala^ genes (referred to as ND2 and adjacent tRNAs, 573 bp), and NADH dehydrogenase subunit 4 (ND4) with adjacent tRNA genes (referred to as ND4 and adjacent tRNAs, 803 bp). We also sequenced the following two nuclear (nuDNA) gene fragments: the recombinase activating gene 1 (RAG-1, 897 bp) and the amelogenin gene (AMEL, 380 bp). The specific PCR primers and conditions for each gene fragment are detailed in Table S3.

Genomic DNA extraction was performed using the TIANamp Genomic DNA Kit (TIANGEN, Beijing, China). Amplified products were visualized on a 1% agarose gel, purified commercially by Sangon Biotech (Beijing, China) and then double-stranded sequenced, using the same primers as in PCR. Chromatograms from both sense and antisense strands were edited and assembled using SeqManII [29] from DNASTAR v5.0 (DNASTAR, Madison, WI, USA) to generate a single, consistent sequence. Sequences were aligned using ClustalW in MEGA v7.0 [30], with manual trimming of partial bases at sequence ends, and equal-length sequences were exported for further analysis. MEGA v7.0 was used to calculate the number of conserved sites (C), parsimony information sites (PI), and variable sites (V) for each mtDNA and nuDNA segment (Table S4.). Haplotypes for concatenated mtDNA were generated using DnaSP v5.10 [31].

### 2.2. Phylogenetic Analyses

We reconstructed the phylogenetic tree of *P. vlangalii* using Bayesian inference (BI) and maximum likelihood (ML) to resolve intraspecific phylogenetic relationships. Following Chen et al. [12], *P. przewalskii* and *P. putjatia* were designated as outgroup taxa (Supplementary Materials, Table S2). The analysis was conducted in PhyloSuite v1.1.15 [32], where all gene sequences (3133 bp) were concatenated using the ‘Concatenate Sequence’ function.

For the BI tree, the ‘Partitionfinder2′ was used to determine the best nucleotide model and partitioning scheme based on the corrected Akaike information criterion (AICc). The BI phylogenetic tree was reconstructed using ‘MrBayes’ [33], with two runs of 20 million generations each, sampling every 1000 generations and discarding the first 25% as burn-in. Nodes with posterior probability (PP) ≥ 0.95 were considered well-supported. The ML tree was generated with ‘IQ-TREE’ [34], using the optimal model determined by AICc for each partition, and node support was assessed using 5000 ultrafast bootstrap approach (UFBoot) replicates; nodes with UFBoot ≥ 95% were considered highly supported [35]. Phylogenetic trees were visualized with FigTree v1.4.4 [36]. A summary of DNA partitions and associated models, as determined by PartitionFinder, is provided in the Supplementary Materials (Table S5).

Haplotype networks for concatenated mtDNA (1856 bp) and nuDNA (1277 bp) alignments were generated separately in PopART v1.7 [37] to illustrate haplotype relationships. Due to the absence of AMEL and RAG-1 sequences in GenBank, only the 130 individuals sequenced in this study were included in the nuDNA analyses. Given the presence of ambiguous sites in the concatenated nuDNA sequence, DnaSP v5.10 [31] was used to generate phased nuDNA alleles.

### 2.3. Divergence Time Estimation

To infer the historical evolutionary processes of *P. vlangalii*, we used Bayesian inference to estimate the divergence times of the major lineages from our concatenated mtDNA dataset through BEAST v1.10.4 [38]. Given the lack of reliable fossil records, our analyses followed a two-step approach similar to other studies [39,40]. In the first step of the analyses, we dated the most recent common ancestor (MRCA) of *P. vlangalii*. We extracted six haplotypes from different clades identified in our previous phylogenetic analyses. In addition, we obtained 15 complete mitochondrial genome sequences of other *Phrynocephalus* species from GenBank (Supplementary Materials, Table S2), with *Laudakia tuberculata* as an outgroup, following Jin and Brown [13]. After sequence alignment, the ‘Partitionfinder2′ function in PhyloSuite was used to select the optimal partition and evolutionary model based on the AICc. Based on the results of Jin and Brown [13], three calibration points were introduced: C1, the MRCA of the genus *Phrynocephalus* [9.78 ± 1.0 Ma (million years ago)]; C2, the MRCA of the viviparous species (5.04 ± 0.5 Ma); and C3, the divergence time of *P. vlangalii*, *P. theobaldi*, and *P. erythrurus* (3.02 ± 0.5 Ma). A relaxed lognormal clock and a birth–death process were used to link clock models and trees. In a second step, we estimated the coalescent time of internal lineages within *P. vlangalii* using the age estimate for its MRCA [2.42 Ma, 95% highest probability density interval (HPD): 1.56–3.20 Ma] as a normal distribution model (mean = 2.42, SD = 0.42). We assumed a relaxed lognormal clock and a constant-size coalescent tree prior. The analysis consisted of 300 million generations of the Markov Chain Monte Carlo (MCMC) chain, with samples taken every 5000 generations. The model specifications, priors, and parameter settings are detailed in the Supplementary Materials (Table S5). We assessed convergence using effective sample sizes (ESS) ≥ 200 in Tracer v1.7.1 [41].

### 2.4. Population Genetic Analyses

We used DNAsp v5.10 [31] to calculate nucleotide diversity (π) and haplotype diversity (*Hd*) for each clade based on our concatenated mtDNA dataset. To evaluate significant genetic differentiation and genetic structure among *P. vlangalii* clades, we used Arlequin v3.5.2.2 [42] to calculate the genetic differentiation coefficient (*F*_st_) and MEGA X [43] to determine the mean genetic distances between and within clades using uncorrected pairwise genetic distances (p-distance).

For a more in-depth analysis of population structure, we performed a spatial analysis of molecular variance (SAMOVA) using SAMOVA v2.0 [44]. In this analysis, populations were divided into distinct groups based on genetic data and geographic distance, with the aim of minimizing genetic variation within groups and maximizing genetic variation between groups, thus facilitating the detection of genetic structure and geographic distribution patterns among populations. The population from Gonghe County (Clade I) was excluded due to limited sampling, resulting in 30 sample sites for analysis. We evaluated *K* values ranging from 2 to 10, with 10,000 simulated annealing processes, to identify the optimal number of groups based on the peak *F*_ct_ values. Groups with fewer than two individuals were not considered.

Following SAMOVA, we used Arlequin to perform an analysis of molecular variance (AMOVA) to estimate the proportion and significance of genetic variation among groups (*F*_ct_), within groups (*F*_sc_), and within populations (*F*_st_) with 100,000 random permutations, following the optimal geographical grouping structure defined by SAMOVA and testing correlations between genetic, environmental, and geographical distances.

We conducted isolation-by-distance (IBD) [45] and isolation-by-environment (IBE) [46] tests to explore the relative impacts of geographic and environmental factors on population differentiation in *P. vlangalii*, based on our concatenated mtDNA dataset.

P-distances between sampling sites were calculated using MEGA X [43]. Geographical distances were estimated with GeographicDistanceMatrixGenerator v1.2.3 [47]. We downloaded 19 contemporary bioclimatic variables from WorldClim v1.4 (http://www.worldclim.org (accessed on 15 August 2023)) [48] and extracted them for our 31 sampling sites using ArcGIS v10.8. After removing highly correlated variables (Pearson′s r > 0.80), we selected seven non-highly correlated environmental variables (Bio1, 2, 3, 7, 12, 15, 17, and 19) for further analyses. Environmental Euclidean distances were calculated using the “vegan” package in R [49]. Mantel tests were performed in ZT v1.1 [50] to assess relationships between genetic distance (*F*_st_/(1 − *F*_st_)) and the logarithm of geographic distance (log km), as well as between genetic distance (*F*_st_/(1 − *F*_st_)) and environmental distance. To eliminate potential spurious correlations between IBD and IBE effects, we conducted partial Mantel tests, controlling for either environmental or geographical distance. Considering the influence of geographical barriers on gene flow, we used the Kunlun–Arjin–Qilian mountains as an indicator for partial Mantel tests, as the distribution of *P. vlangaii* has not crossed the northern peaks of the QTP. The test results were visualized using Microsoft Excel 2021.

### 2.5. Inference of Demographic Histories

We inferred the historical population size fluctuations for the major clades of *P. vlangalii* using three different approaches with concatenated mtDNA data. First, we calculated Tajima’s *D*, Fu’s *Fs*, and *R*_2_ statistics and their respective *p*-values in DnaSP, with 10,000 simulated samples [31]. These tests provide insight into the historical dynamics of the population under various scenarios. Tajima’s *D* indicates long-term population events, while Fu’s *Fs* and *R*_2_ are sensitive to recent population expansions. Fu’s *Fs* is particularly suitable for large sample sizes, while *R*_2_ is more appropriate for smaller samples. Second, we generated a mismatch distribution (MD) plot using Arlequin for each *P. vlangalii* clade. To assess hypotheses of recent population growth, we estimated the sum of squared deviations (*SSD*) and Harpending’s raggedness index (*Rg*) with 10,000 bootstrap replicates. If the frequency distribution of alleles is relatively uniform, the *Rg* value is lower; if it exhibits significant skewness or unevenness, the *Rg* value is higher. Finally, we constructed Bayesian skyline plots (BSPs) [38] using BEAST to model changes in population size over time. The nucleotide substitution model was determined using ‘PartitionFinder2′ in PhyloSuite v1.1.15 [32]. A strict clock model was applied with a substitution rate of 1.15%/site/million years (95% HPD: 0.47–1.92%/site/million years), based on previous divergence dating analysis. The model specifications, priors and parameters are detailed in Table S5. The results of each analysis were visualized using Tracer.

### 2.6. Morphology Data Collection and Analyses

We examined 82 newly sampled *P. vlangalii* specimens from the NKAQ and obtained 13 meristic characters and 15 metric characters (Table S6), which are traditionally used to diagnose *Phrynocephalus* species [21,24,51]. Collection methods and definitions of diagnostic characters followed those of Zhao et al. [21]. Specimens were sexed by inspecting the reproductive organs, and individuals with a snout–vent length (SVL) greater than 45 mm were classified as adults, while smaller individuals were classified as juveniles [52]. Metric characters were measured to the nearest 0.01 mm using a digital caliper, and meristic characters were assessed using a binocular microscope.

To determine if *P. vlangalii* specimens occupied different positions in morphospace and to validate the molecular phylogenetic results, we conducted a principal component analysis (PCA) using SPSS version 19 (SPSS Inc., Chicago, IL, USA). Meristic and metric characters were analyzed separately due to the lack of known correlations. As no sexual dimorphism or adult–juvenile differences were observed in meristic characters, all individuals were included in a single PCA. However, as sexual dimorphism affects metric characters [51], males and females were analyzed separately in the PCA for these characters.

### 2.7. Ecological Niche Modeling

To explore potential range shifts for *P. vlangalii* from the Pleistocene to the 2070s under climate change scenarios, we employed Ecological Niche Modeling (ENM) with MaxEnt v3.4.1 [53]. MaxEnt is a widely used method for predicting species distributions, particularly with presence-only data, and it can project range changes under various climatic conditions.

Occurrence data, comprising 69 localities, were compiled from fieldwork and previous studies with precise coordinates (Table S7). To reduce spatial bias, we used SDMtoolbox v2.5 [54] in ArcGIS to filter the dataset, ensuring that no two localities were closer than 10 km, resulting in 56 localities. The study area was defined by a minimum bounding rectangle around the localities with a 5° buffer [55,56], covering the known range of *P. vlangalii* from 76 to 107° E and from 28 to 44° N.

We obtained 19 climate variable factors for the following five different time periods from the WorldClim database (http://www.worldclim.org (accessed on 15 August 2023)) [48]: the present (1960–1990), the last interglacial (LIG, 0.12–0.14 Ma), the Last Glacial Maximum (LGM, ~0.02 Ma), the middle Holocene (MH, ~6000 years ago), and the future 2070s (2061–2080). The LGM data had a resolution of 2.5 arc-minutes, while the others were all 30 arc-seconds. We chose the Model for Interdisciplinary Research on Climate (MIROC) for the LGM and MH and applied it under the Shared Socio-economic Pathways 126 and 585 (SSP 126/585) for the 2070s, representing the lowest and highest carbon emission situations.

We estimated correlations between the 19 contemporary climate variable factors using SDMtoolbox [54] in ArcGIS, retaining variables with low correlation (r < 0.8). Eight variables (Bio1, 2, 3, 7, 12, 14, 15, and 19) were selected for analysis (Supplementary Materials, Table S8). The current distribution model was based on contemporary climate data, with niche models for other periods projected from the contemporary dataset. MaxEnt randomly allocated 70% of the data for training and 30% for testing, with 100 bootstrap replicates.

The ENMeval package [57] in R was used to manage model complexity and to determine the optimal MaxEnt feature classes and regularization multipliers. The optimal model had a multiplier of 0.1 and a linear/quadratic feature class, with other parameters set by default. To avoid over-extrapolation, we conducted a multivariate environmental similarity surface (MESS) analysis [58] to check for combinations of climatic variable not represented in the training data, indicating reliable or unreliable projection areas. Model reliability was evaluated using the area under the receiver operating characteristic curve (AUC), with AUC > 0.7 indicating a fair model. The habitat suitability index ranged from 0 to 1, with higher values indicating greater suitability. We used maximum training sensitivity plus specificity (MTSS) and balanced training omission, predicted area, and threshold value (BTPT) as classification thresholds, dividing the habitat suitability index into highly, secondarily, and unsuitable habitats (BTPT = 0.0237, MTSS = 0.1946). The importance of each climate variable was assessed based on the contribution values reported in the MaxEnt output files.

## 3. Results

### 3.1. Phylogenetic Relationships

**Figure 2:**
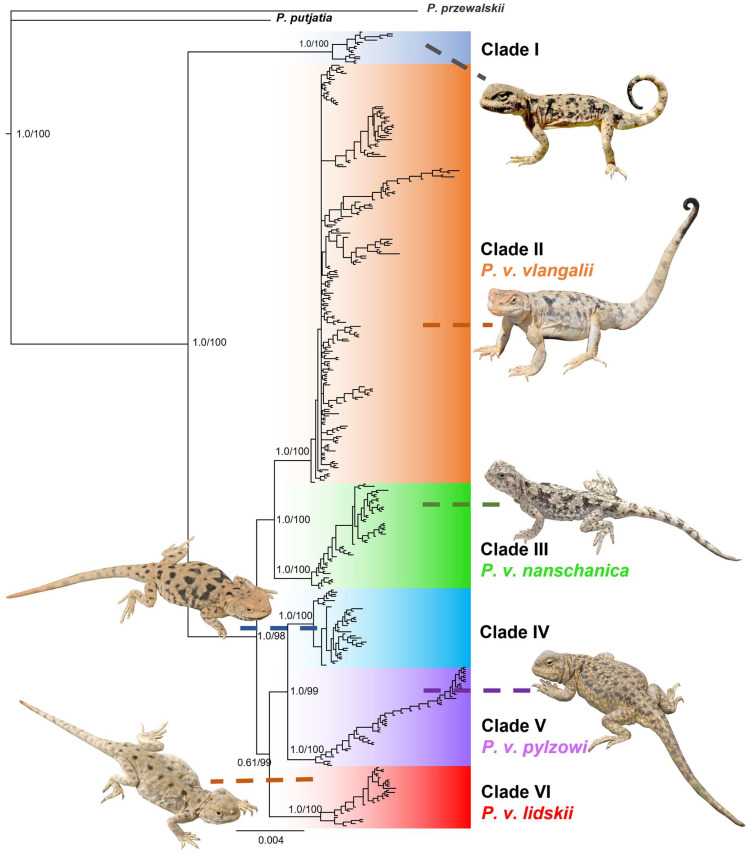
A 50% majority-rule consensus tree inferred from the whole concatenated sequence (3133 bp) using Bayesian inference with MrBayes v.3.2, with 2 × 10^7^ generations for BI and 5000 UFBoot replicates for ML. Six clades are identified within *P. vlangalii*, each highlighted for visual distinction. Bayesian posterior probability (PP) and maximum likelihood UFBoot values are shown at major nodes. Photo of Clade I by Jian Liu; photos of clades II, IV, and VI by Xianguang Guo; and photos of clades III and V by Zhongyi Yao.

Both BI and ML analyses revealed a consistent tree topology, indicating that individuals of *P. vlangalii* form six well-differentiated clades with high support values from PP and UFBoot, respectively (Figure 2). These clades correspond well to distinct geographical distribution ranges, which are strongly supported by the data (Figure 1). Individuals from Gonghe County, Qinghai Province (L28), were the first to diverge, forming an independent genetic lineage, Clade I (PP = 1.0, UFBoot = 100), in agreement with Chen et al. [12]. With the exception of L28, *P. vlangalii* from other localities formed a monophyletic group (PP = 1.0, UFBoot = 98). Within this group, clades II and III formed a well-supported cluster (PP = 1.0, UFBoot = 100), while a second cluster comprising clades IV, V, and VI showed unresolved relationships (PP = 0.61, UFBoot = 99). Clade II, corresponding to the subspecies *P. v. vlangalii*, included individuals from the Qaidam Basin (L11,16–27), Sugan Lake (L12), and Ruoqiang County (L9,10) (PP = 1.0, UFBoot = 100). Clade III, comprising *P. v. nanschanica*, consisted of individuals from the North Qilian Mountains, including Akesai County (L13) and Subei County (L14,15) in Gansu Province (PP = 1.0, UFBoot = 100). Clade V included populations from the headwaters of the Yellow River, such as Maduo County and Maqu County in Qinghai Province, and Ruoergai County in Sichuan Province, representing the southernmost distribution of *P. vlangalii* and the subspecies *P. v. pylzowi*. Phylogenetic analysis also divided *P. vlangalii* individuals from Xinjiang into two clades. Clade IV, an undescribed new lineage, included populations from Minfeng County and Qiemo County. Clade VI, which evolved independently, should belong to the subspecies *P. v. lidskii*, according to Peters [25], since its type locality is exactly Yutian County, where this clade is distributed.

**Figure 3:**
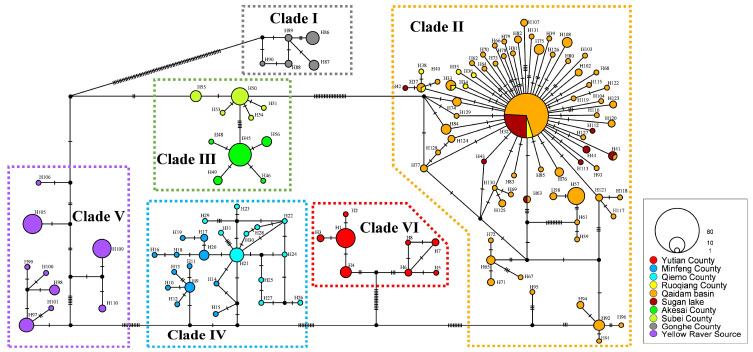
TCS network of haplotypes inferred from a total of 382 concatenated mtDNA sequences for *P. vlangalii* using PopART v1.7 software. Each colored circle represents a single haplotype; black dots indicate inferred vectors. Different filled colors correspond to the geographical origins of the haplotypes. The size of each circle is proportional to the number of individuals sharing that particular haplotype. Short bars crossing haplotypes represent substitution steps and indicate the genetic distance between them.

For the haplotype networks, one sample, identified by the voucher number Guo5226, was excluded from this part of the analyses due to unsuccessful sequencing of the ND4-tRNA^Leu^ gene. Based on the remaining mtDNA dataset of 382 individuals, we identified a total of 131 haplotype sequences and constructed a haplotype network using the method of Templeton, Crandall and Sing (TCS) [59,60] (Figure 3). The network revealed six major haplogroups, consistent with the topology of the phylogenetic tree. These haplogroups exhibit high differentiation and geographic specificity among *P. vlangalii* populations. Each clade has several dominant haplotypes, with Clade II showing a distinct star-like network centered on the most abundant haplotype (H32, comprising 79 sequences), suggesting a recent population expansion.

For nuDNA, we obtained 86 haplotype sequences after excluding Clade I (Gonghe County lineage), Clade V (Yellow River headwaters, *P. v. pylzowi*), and part of Clade II (Qaidam Basin, *P. v. vlangalii*) from the mtDNA phylogenetic tree. The haplotype network was reconstructed using the median-joining method [61]. Due to the conservation of nuclear gene sequences, the mtDNA-based topological structure was not well-supported in the nuDNA haplotype network (Supplementary Materials, Figure S1). Additionally, common haplotypes were observed between different clades, such as between Clade II and Clade III, and between Clade IV and Clade VI.

### 3.2. Divergence Times Estimation

**Figure 4:**
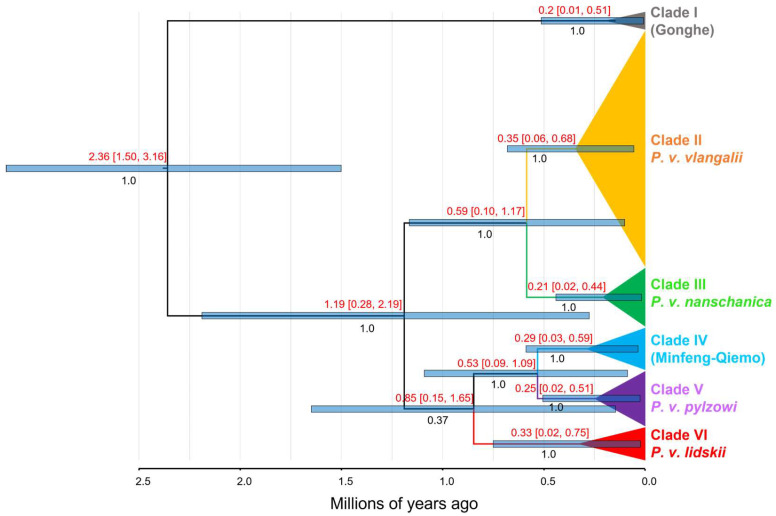
Molecular dating of *P. vlangalii*, based on the concatenated mtDNA. Divergence dates with 95% HPD and posterior probability for major nodes are shown.

The first analysis of divergence times within the genus *Phrynocephalus*, as depicted in Figure S2, indicates that *P. vlangalii* diverged from other *Phrynocephalus* species during the Pliocene epoch (~2.55 Ma, 95% HPD: 1.78–3.39 Ma). The MRCA of *P. vlangalii* was dated to the early Pleistocene (~2.36 Ma; 95% HPD: 1.50–3.16 Ma), as shown in Figure 4. The earliest divergence within *P. vlangalii* was identified as Clade I, the population from Gonghe County. The remaining *P. vlangalii* populations split into two major clusters around 1.19 Ma (95% HPD: 0.28–2.19 Ma). One of these clusters included clades II and III, which diverged around 0.59 Ma (95% HPD: 0.10–1.17 Ma). Among the remaining clades, clades IV and V diverged approximately 0.53 Ma (95% HPD: 0.09–1.09 Ma). Support for the differentiation of Clade VI relative to other clades is low (PP = 0.37), and its estimated time of divergence from other branches is around 0.85 Ma (95% HPD: 0.15–1.65 Ma).

### 3.3. Genetic Diversity and Genetic Structure

Excluding the sample Guo5226, a total of 126 haplotypes were defined from the concatenated mtDNA dataset of 382 individuals of *P. vlangalii*. The overall genetic diversity of *P. vlangalii* is characterized by high haplotype diversity (0.944) and low nucleotide diversity (0.01424) (Table 2). Haplotype diversity varied among the six clades, ranging from a low of 0.709 (Clade IV) to a high of 0.938 (Clade IV). Nucleotide diversity also varied among the clades, with the lowest value of 0.001003 observed in Clade I and the highest value of 0.00413 in Clade II.

The SAMOVA analysis of *P. vlangalii*, excluding Clade I, showed that the *F*_ct_ value increased with the number of groups (*K*) increased from two to ten. However, when *K* reached six or more, some populations were merged into single groups. This, *K* = five, was identified by SAMOVA as the optimal number of groups, in agreement with the results of the phylogenetic analysis. The *F*_ct_ values for *K* = two, three, four, and five were 0.5047, 0.6363, 0.7485, and 0.8452, respectively.

The AMOVA analysis (Table 3), based on the SAMOVA grouping, showed significant genetic differences mainly between groups when the 30 populations were divided into two, three, four, and five groups (50.47%, 63.63%, 74.85%, 84.52%), with highly significant genetic differentiation coefficients (*p* < 0.01).

The genetic differentiation coefficient *F*_st_ calculated between the six clades of *P. vlangalii* is shown in Table 4. The *F*_st_ values ranged from 0.75579 (between clades IV and V) to 0.97079 (between clades I and VI), indicating substantial genetic differentiation between each pair of clades. All significance tests were highly significant (*p* < 0.01), underscoring the genetic distinctiveness among the clades.

The p-distances between and within the clades are presented in Table 4. The average inter-clade genetic distance ranged from 1.1% (between clades IV and V) to 4.4% (between clades I and VI), with no pair exceeding a distance of 5%. Intraclade genetic distances varied from 0.1% (within Clade I and Clade VI) to 0.3% (within Clade V).

### 3.4. IBD and IBE

The results of the IBD and IBE analyses for *P. vlangalii* are detailed in the Supplementary Materials (Figure S3). The IBD analysis, the Mantel test, revealed a highly significant positive correlation between genetic distance and geographic distance among populations (*R*_2_ = 0.1938, *p* < 0.001), supporting the IBD model. In the partial Mantel test, when controlling for environmental barriers, a significant positive correlation between genetic and geographic distance was observed (*r* = 0.2697, *p* < 0.01). Conversely, when controlling for geographic distance, a significant positive correlation was found between genetic distance and geographical barriers (*r* = 0.2215, *p* < 0.05), indicating that both geographic distance and environmental factors, such as the Kunlun–Arjin–Qilian Mountains, significantly influence population differentiation in *P. vlangalii*.

For the IBE analysis, the Montel test showed a significant positive correlation between genetic distance and environmental distance (*R*_2_ = 0.3996, *p* = 0.002), consistent with the IBE model. In the partial Montel test, when controlling for geographic distance, a significant positive correlation was observed between genetic and environmental distance (r = 0.2468, *p* < 0.05). Similarly, when controlling for environmental distance, a significant positive correlation was found between genetic and geographic distance (r = 0.3146, *p* < 0.05). The results suggest that both environmental and geographic distances contribute to population differentiation in *P. vlangalii*, with a stronger correlation observed between geographic and genetic distances.

### 3.5. Historical Demographic Change

The population expansion tests for each clade of *P. vlangalii*, including Tajima’s *D*, Fu’s *Fs*, and *R*_2_ values, are presented in Table 5. For Clade II, both Tajima’s *D* and Fu’s *Fs* are highly significantly negative, while *R*_2_ is highly significantly positive. These results indicate a strong signal of population expansion for Clade II. In the case of Clade IV, Tajima’s *D* is not significantly negative, but Fu’s *Fs* is highly significantly negative and *R*_2_ is also significantly positive. This suggests that Clade IV has also experienced some degree of population expansion, although the signal is not as strong as for Clade II.

**Figure 5:**
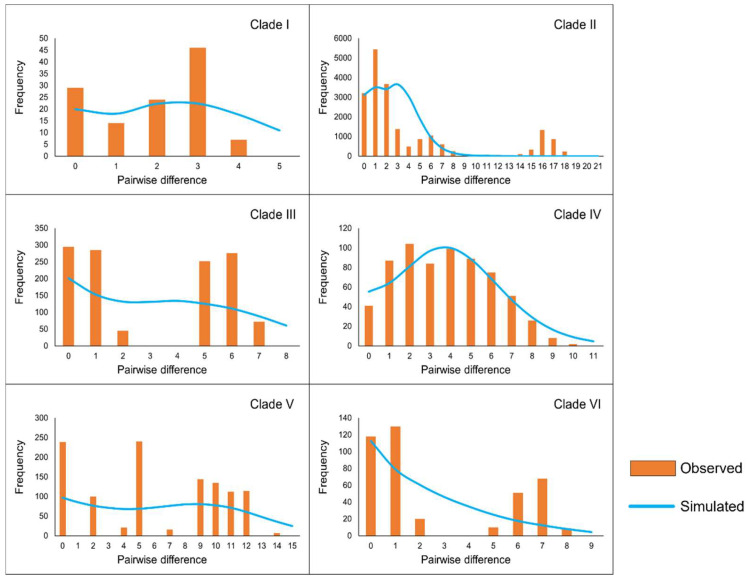
Results of the mismatch distribution analysis for each clade. Orange bars represent the pairwise differences in the observed distribution, and blue lines represent the theoretically expected distribution under a population expansion model.

Analysis of the mismatch distribution (Figure 5) revealed that Clade II and Clade IV exhibited unimodal curves, a characteristic often associated with populations that have undergone rapid expansion. Furthermore, the *SSD* and *Rg* values for these clades were small positive numbers, which are not significantly different from zero, further supporting the inference of a historical population growth.

**Figure 6:**
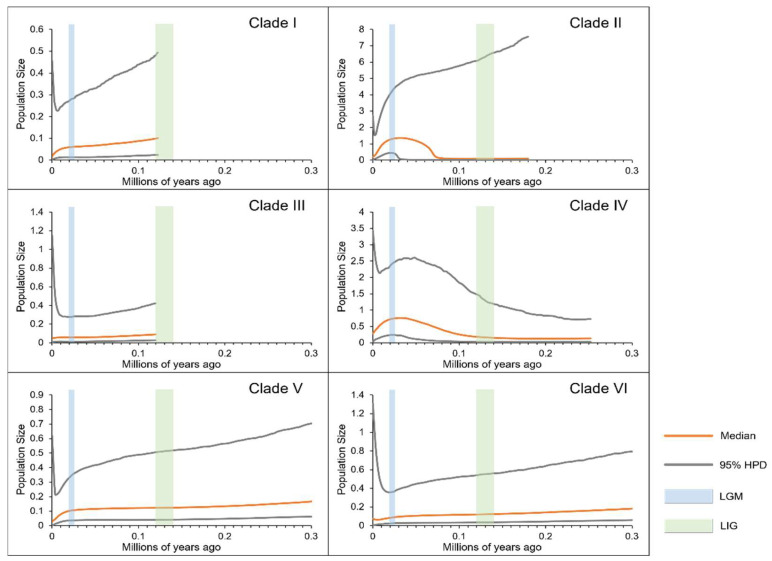
Bayesian skyline plots for each clade. The y-axes represent the estimated effective population size on a logarithmic scale (Ne*τ/10^6^, the product of the female effective population size and generation length in years); the x-axes represent time in millions of years ago. The vertical blue and green bars represent the duration of the Last Glacial Maximum (LGM) and the Last Interglacial (LIG), respectively.

The BSP results for each *P. vlangalii* clade, as shown in Figure 6, indicate that Clade II and Clade V underwent a rapid expansion after the LIG, followed by contraction after the LGM. These patterns are consistent with the results of our neutrality tests and mismatch distribution analyses. In contrast, the population sizes of the other clades remained relatively stable in the past and only experienced only minor contractions after the LGM.

### 3.6. Morphological Diversity

**Figure 7:**
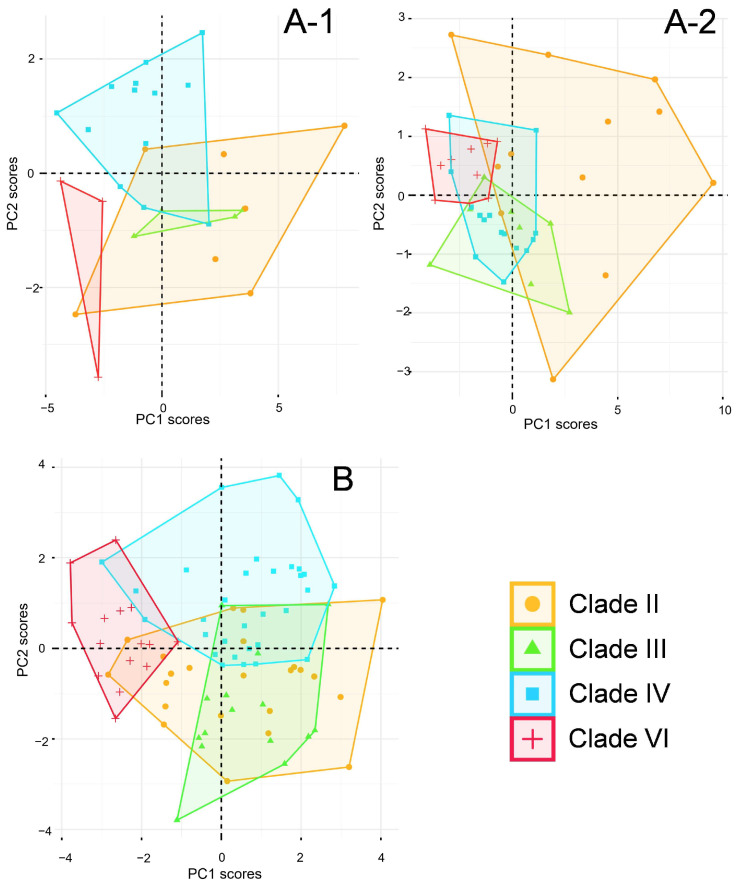
Result of PCAs for each clade of *P. vlangalii*. (**A**), metric characters (**A**-1 for male; **A**-2 for female); (**B**), meristic characters.

All morphological data collected for *P. vlangalii* are detailed in Supplementary Materials (Table S9). Principal component analyses (PCAs) of metric traits for both males and females extracted three principal components from the morphological variables, as detailed in Supplementary Materials (Table S10). For the first principal component (PC1), all metric traits show significant loadings (>0.4), accounting for 58.5% of the variance in males and 63.0% in females, indicating that PC1 is a reasonable representation of individual size. PC2 shows sexual dimorphism in *P. vlangalii*. In males, high loadings for traits such as tail length (TL), head length (HL), head height (HH), forelimb length (FLL), hind limb length (HLL), and fourth toe claw length (4th TCL) account for 12.4% of the variance. For females, PC2 is mainly loaded on HL and 4th TCL, with less clear patterns, explaining only 6.7% of the variance. The PCA results are shown in Figure 7A, where Clade II occupies the largest area and overlaps with other clades. Male Clade VI shows minimal overlap with other clades for metric traits, while female Clade VI has limited overlap with Clade III, and Clade IV overlaps strongly with both Clade VI and Clade III.

For meristic traits, four principal components were extracted from the PCAs of all individuals (Supplementary Materials, Table S10). The PC1 includes significant loadings for scale length (SL), interlimb length (IL), scale midline gap (SMG), dorsal openings (DO), ventral edges (VE), subauricular muscles (SAM), and fourth toe intermediate scale (4thTI), accounting for 26.2% of the variance. PC2, PC3, and PC4, mainly composed of head scales, account for 16.0%, 9.0%, and 8.0% of the variance, respectively. The PCA results for the meristic characters are shown in Figure 7B. Clade VI shows limited interaction with Clade III and other clades, indicating substantial differences. Most individuals in Clade III overlap with Clade II, while half of the individuals in Clade IV overlap with other clades, suggesting relatively minor differences.

### 3.7. Temporal Changes of Suitable Distributional Areas

In our analysis, the three climate variables that contribute most to the distribution of *P. vlangalii* are Bio1 (annual mean temperature, accounting for 48%), Bio2 (mean diurnal range, 24.3%), and Bio7 (temperature annual range, 7%). These results underscore the importance of temperature as the primary factor influencing the potential geographical distribution pattern of *P. vlangalii* (see Supplementary Materials, Table S8). The response curves between the environmental variables and the predicted probability of presence predominantly exhibit non-linear relationships (Supplementary Materials, Figure S3). The probability of presence peaks at 2.5 °C for Bio1, 15.2 °C for Bio2, and 41 °C for Bio7, respectively, are consistent with the high-latitude climate pattern.

The AUC values for the training and test datasets were 0.963 ± 0.006 and 0.958 ± 0.014, respectively, indicating the excellent credibility of the simulation results.

**Figure 8:**
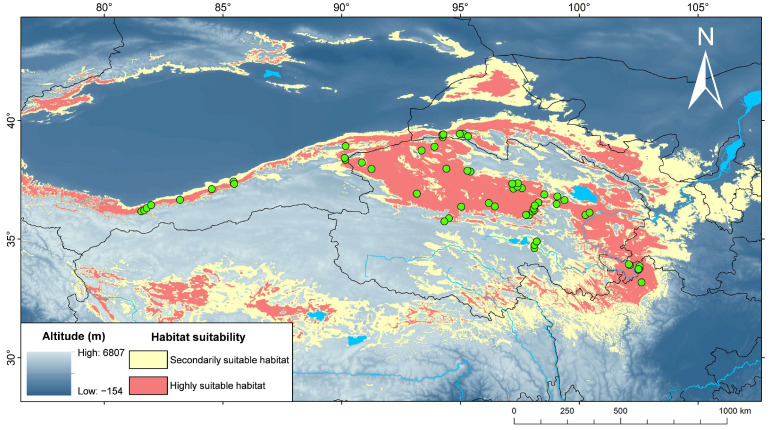
Predicted suitable range at contemporary era for *P. vlangalii*. Green dots represent occurrence data used in Maxent. Yellow and red areas indicate suitable habitat.

**Figure 9:**
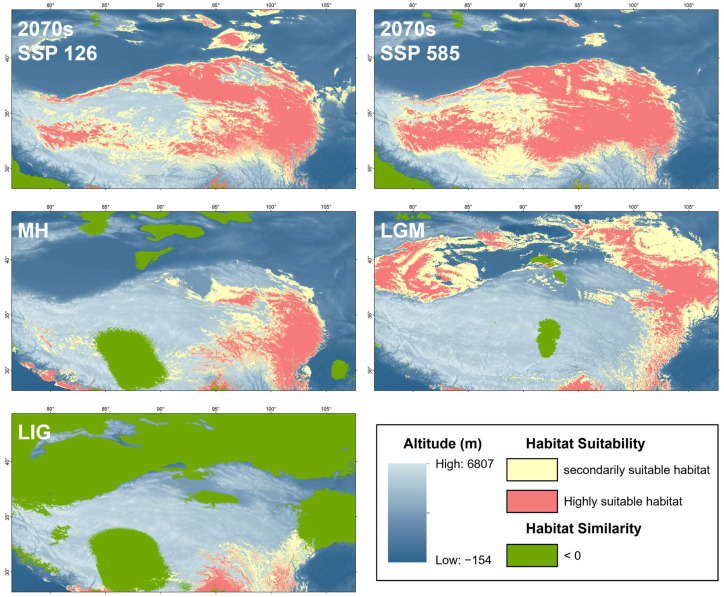
Potentially suitable range for *P. vlangalii* in five different time periods. Green area represents MESS scores < 0, indicating areas with no current equivalent of climatic conditions.

Currently (as shown in Figure 8), the highly suitable and the secondarily suitable habitats for *P. vlangalii* are widely distributed throughout our study area. In particular, the most important predicted suitable habitats include the NKAQ, the Qaidam Basin, the Yellow River headwaters, the Hexi Corridor, Nagqu and Ngari Prefecture in Tibet, and the area south of Tianshan Mountain. The predicted suitable habitat areas for all periods are detailed in Table 6. In the LIG (Figure 9), *P. vlangalii* occupied the area south of the QTP, with the smallest suitable habitat area under significant climate differences from the present. Afterwards, however, a rapid expansion occurred, and *P. vlangalii* reached its largest suitable habitat area in the past during the LGM, with the suitable habitat mainly on the periphery of the QTP. The suitable habitat then contracted prior to the MH. During the MH, the suitable habitat was mainly distributed in the Qaidam Basin and eastern QTP. These changes in the area are consistent with the results of previous demographic analyses. In the 2070s, the suitable habitat for *P. vlangalii* is expected to expand again, shifting to higher elevations of the QTP, especially under the SSP 585 scenario.

## 4. Discussion

### 4.1. Evolutionary History of P. vlangalii

Since the Quaternary, the uplift of the Qinghai–Tibet Plateau, aridification in the western region, and the cyclical glaciations have profoundly influenced the evolution of reptiles. These factors have driven recent and rapid diversification through various mechanisms. This is exemplified by the Tibetan toad-headed agama, *P. theobaldi,* a close relative of *P. vlangalii* [62].

Based on a phylogenetic tree constructed from three mtDNA and two nuDNA markers, the entire *P. vlangalii* species was divided into six distinct clades, each with strong statistical support. A study by Chen et al. [12] explored the reasons for multiple divergence nodes through the analysis of molecular clocks and population barriers. The earliest differentiation within *P. vlangalii* was identified as the Gonghe County population. Chen et al. [12] proposed that this differentiation corresponds to the uplift of the Elashan Mountains during the second phase of the Qingzang Movement (~2.5 Ma), which was the most significant uplift phase. Subsequently, the remaining *P. vlangalii* split into two major branches, clades II + III and clades IV + V + VI, associated with the uplift of the Anyemanqen Mountains during the Kunhuang Movement (1.1–0.6 Ma).

Subsequently, the differentiation of the subspecies *P. v. vlangalii* and *P. v. nanschanica* was attributed to the uplift of the Arjin Mountains. We largely agree with the views of Chen et al. [12]. However, their study lacked strong posterior probability support for the differentiation time of *P. v. vlangalii* and *P. v. nanschanica*, possibly due to an insufficient number of haplotypes obtained for *P. v. nanschanica* at that time. Our analysis determined the differentiation time of *P. v. vlangalii* and *P. v. nanschanica* to be approximately 0.59 Ma (95% HPD: 0.10–1.17 Ma), with a high posterior probability support (PP = 1.0), confirming that the uplift of the Arjin Mountains during the Kunhuang Movement blocked the gene flow of *P. v. vlangalii* and *P. v. nanschanica* [12].

For the other three clades, the phylogenetic tree and molecular clock constructed by Bayesian inference did not fully resolve the phylogenetic relationships between the Minfeng–Qiemo populations, *P. v. pylzowi* and *P. v. lidskii* (PP < 0.9). The time of differentiation of *P. v. lidskii* and other clades is estimated to be around 0.85 Ma, coinciding with the period of the Kunhuang Movement when the Kunlun Mountains underwent significant uplift, possibly causing differentiation [5]. The Minfeng–Qiemo population and *P. v. pylzowi* diverged around 0.53 Ma, close to the Kunlun glacial period in China (0.78–0.56 Ma [63]). During this period, the higher elevations of the QTP were covered by ice sheets, possibly causing *P. vlangalii* populations to migrate to lower elevations. It is hypothesized that multiple glacial refugia on the QTP blocked gene flow between the two clades, leading to differentiation [64].

Furthermore, our neutral analysis, mismatch distribution, and BSP all indicated significant population expansion in clades II and IV, consistent with the Glacial Maximum (GM) expansion model [65]. Additionally, the ENM analysis results revealed a larger area of suitable habitat during the LGM. During this period, the dry and cold climate affected a large area of desert and sandy areas in northern China, intensifying sand-drift activity and increasing desert areas, which may be directly responsible for the expansion of *P. vlangalii* [66].

### 4.2. Genetic Structure of P. vlangalii from the NKAQ

In this study, we identified four clades within *P. vlangalii* from the NKAQ region, each with a distinct regional structure. Clade II (Ruoqiang County), Clade III (Akesai and Subei counties), and Clade V (Yellow River headwaters) correspond to the subspecies *P. v. vlangalii*, *P. v. nanschanica*, and *P. v. pylzowi*, respectively. The population from the northern Kunlun Mountains was first described by Bedriaga [18] as a new variant of *P. v.* var *lidskii*. However, Bedriaga’s original description was based on syntype specimens without specifying a type locality. Peters [25] later identified the type locality in the Keria Mountains, Yutian County. Our phylogenetic analysis revealed that the range of *P. v.* var *lidskii* includes two strongly supported monophyletic groups, Clade IV and Clade VI, representing the Minfeng–Qiemo and the Yutian populations, respectively. Following the principle of priority, we have designated the Yutian population as *P. v. lidskii*, while the Minfeng–Qiemo population is recognized as a cryptic new lineage.

The p-distances and *F*_st_ values between the concatenated mtDNA of different known subspecies of *P. vlangalii* range from 0.11 to 0.44 and from 0.75579 to 0.97079, respectively. Despite the close geographical proximity of the Minfeng–Qiemo and Yutian populations, the genetic distance between them is 0.016, and the *F*_st_ value is 0.89296, indicating that the genetic differentiation has reached the subspecies level. This suggests that Clade IV, distributed in Minfeng County and Qiemo County, should be recognized as a new subspecies of *P. vlangalii*.

In our analysis of the nuDNA haplotype network, we did not observe a clear geographical structure as in the case of mtDNA. This phenomenon has been observed in other species, such as the variegated racerunner (*Eremias vermiculata*), the Barcheck darter (*Etheostoma obeyense*), and the dry forest toad (*Incilius coccifer*) [67,68,69]). There are several potential explanations for the discrepancies between nuDNA and mtDNA, including the following: (i) incomplete lineage sorting (ILS); (ii) gene flow between lineages; (iii) sex-biased dispersal; and (iv) populations undergoing secondary contact [70,71]. Male-biased dispersal has been confirmed in *P. vlangalii*, with females exhibiting philopatry and males having a wider range of activity [72]. This male-mediated gene flow may reduce differences in nuDNA structure between regions.

Additionally, *P. vlangalii* lineages may be influenced by ILS. NuDNA has a lower rate of base substitution than mtDNA, and the differentiation time between lineages is relatively short, leading to the random fixation of mutations and the sharing of ancestral polymorphisms between these lineages.

### 4.3. Morphological Diversity of P. vlangalii from the NKAQ

The different clades of *P. vlangalii* in the NKAQ have unique morphological characteristics. *P. v. lidskii*, which inhabits the desert and clay habitats of Yutian County, has a sandy yellow body color that is well camouflaged in its environment. This subspecies lacks a prominent dorsal midline and has several pairs of distinct black spots on either side of the spine, extending from the nape to the tail base. The first pair of black spots behind the forelimbs may extend outwards in a dumbbell shape, and a slightly lighter cyan pattern is present on the back, excluding the black spots. *P. v. lidskii* is the smallest of the four clades, with an average snout–vent length (SVL) of 48.63 mm (SD: 2.28 mm), almost 10 mm shorter than the largest subspecies, *P. v. vlangalii*, which has an average SVL of 57.53 ± 8.21 mm. The largest individual of *P. v. lidskii* in this study measured only 51.64 mm.

The Minfeng–Qiemo population has a similar body color to *P. v. lidskii* but is slightly larger, with an average SVL of 50.46 ± 2.75 mm. Notably, this population typically has a tail length (TL) that is equal to or greater than its SVL, averaging 54.44 ± 4.59 mm. This characteristic distinguishes it from other clades, which generally have a TL that is shorter than the SVL. The Minfeng–Qiemo population also shows the highest ratios of forelimb length (FLL), hind limb length (HLL), SMG, number of ventrals (VE), and fourth toe infratarsals (4th TI) to SVL, suggesting a high adaptability to sandy environments.

There are significant morphological differences between populations of *P. v. vlangalii* from different regions [73], with the NKAQ population being the largest in body size. The Sugan Lake population is particularly notable, with the largest individual reaching an SVL of 73.71 mm (a female). However, this clade has the lowest ratios of TL, FLL, HLL, and distance across the axilla and groin (DAG) to SVL, even significantly smaller than other clades with a stout trunk.

*Phrynocephalus v. nanschanica* shows no significant differences from other clades in principal component analysis (PCA) for either metric or meristic characters. Its back is grayish-white with large gray or yellowish spots extending to the sides of the body. The spots are irregularly shaped with a black border. Numerous small black dots, each consisting of one or a few scales, are present on the back, limbs, and tail, contrasting sharply with the background color.

In both carbon emission scenarios, *P. vlangalii* is projected to experience some expansion of its total suitable habitats by the 2070s. However, the ranges of most of its clades are relatively limited, and differentiation in morphology implies differentiation in ecological niches. It is therefore imperative to consider conservation strategies for taxonomic units below the species level.

Fortunately, the recently established Qilian Mountain National Park and Kunlun Mountain National Park will cover most of the range of *P. vlangalii* in the NKAQ. Conservation efforts for this species will also be of great importance to the establishment and development of the two national parks.

## 5. Conclusions

This study provides a focused yet comprehensive examination of the phylogeography and diversity of the Qinghai toad-headed agama (*P. vlangalii*) across the Qinghai–Tibet Plateau. The identification of six distinct genetic lineages highlights the central role of geological and glacial history in shaping the evolution of the species. The observed high genetic diversity and significant divergence between clades, influenced by geographic and environmental factors, highlight the need for conservation strategies that take into account fine-scale spatial heterogeneity. The morphological data are consistent with the genetic results, further highlighting the adaptability of *P. vlangalii* to the geological and climatic changes in the region. The resilience of the species is evidenced by the lack of population decline during the Last Glacial Maximum and the anticipated future habitat expansion, demonstrating its ability to adapt to environmental change. Taken together, our research highlights the critical importance of integrating geological and climatic factors into conservation efforts, and emphasizes the need for ongoing research to safeguard the species against the backdrop of ongoing environmental change.

## Figures and Tables

**Table 2:** Summary statistics of genetic diversity for each clade in *P. vlangalii*.

Clade	*n*	*H*	*Hd*	*π*
I	16	5	0.758 ± 0.080	0.00103 ± 0.00011
II	218	73	0.855 ± 0.023	0.00413 ± 0.00046
III	50	10	0.759 ± 0.045	0.00167 ± 0.00011
IV	37	23	0.938 ± 0.027	0.00202 ± 0.00021
V	48	22	0.788 ± 0.032	0.00332 ± 0.00019
VI	29	8	0.709 ± 0.081	0.00143 ± 0.00030
Total	382	126	0.944 ± 0.008	0.01424 ± 0.00066

**Table 3:** Results of AMOVA for *P. vlangalii* other than Clade I.

Hypothesized Structure	Among Groups			Between Populations			Among Populations		
	*df*	%var	*F*_ct_	*df*	%var	*F*_sc_	*df*	%var	*F*_st_
No structure				29	93.96		336	6.04	0.93957
*K* = 2	1	50.47	0.50470	28	46.07	0.93018	336	3.46	0.96542
*K* = 3	2	63.63	0.63526	27	32.30	0.88808	336	4.07	0.95929
*K* = 4	3	74.85	0.74854	26	20.66	0.82160	336	4.49	0.95514
*K* = 5	4	84.52	0.84518	25	11.11	0.71769	336	4.37	0.95629

The genetic differentiation coefficients are all extremely significant.

**Table 4:** The mean uncorrected pairwise distance (p-distance) within each clade (bold diagonal) and between clades (bottom diagonal), together with the pairwise genetic differentiation *F*_st_ and its significance between clades (top diagonal) for *P. vlangalii*.

Clade	I	II	III	IV	V	VI
I	0.001	0.94773 *	0.96367 *	0.95824 *	0.93373 *	0.97079 *
II	0.042	0.002	0.85091 *	0.86520 *	0.86438 *	0.88265 *
III	0.042	0.015	0.002	0.89136 *	0.86554 *	0.92031 *
IV	0.041	0.017	0.017	0.002	0.75579 *	0.89296 *
V	0.041	0.019	0.018	0.011	0.003	0.86559 *
VI	0.044	0.019	0.020	0.016	0.019	0.001

Significance level: 0.01 *.

**Table 5:** Neutrality tests and the indicators of mismatch distribution for each clade of *P. vlangalii*.

Clade	Tajima’s *D*	Fu’s *Fs*	*R*_2_	*SSD*	*Rg*
I	0.85989	0.18164	0.15691	0.05819	0.16521
II	−2.27806 **	−25.42324 **	0.08047 **	0.01726	0.03982
III	0.20213	−0.19232	0.10647	0.07700	0.11369
IV	−1.37197	−15.39601 **	0.11797 *	0.00321	0.01103
V	0.78254	3.73030	0.10731	0.07546 **	0.17139 **
VI	0.48321	−0.41435	0.12636	0.06625	0.11087

Significance level: 0.05 *; 0.01 **.

**Table 6:** The area of predicted suitable habitat for each period of *P. vlangalii*.

Period	Highly Suitable Habitat (km^2^)	Secondarily Suitable Habitat (km^2^)	Total Suitable Habitat (km^2^)
Present	45.55535	75.47528	121.03063
LIG	11.06444	23.41674	34.48118
LGM	64.07291	94.32986	158.40277
MH	41.35229	50.57611	91.92840
2070s-126	86.65292	83.99479	170.64771
2070s-585	115.08100	78.94542	194.02642

## Data Availability

The data supporting the results of this study can be found in the manuscript. All sequences generated during this study have been deposited in GenBank (https://www.ncbi.nlm.nih.gov/genbank/ (accessed on 8 October 2024)) under accession numbers PQ433898–PQ434416 and PQ436674–PQ436803.

## Supplementary Materials

The following supporting information can be downloaded at: https://www.mdpi.com/article/10.3390/ani15030400/s1, Figure S1: Median-joining network of haplotypes inferred from a total of 130 concatenated nuDNA sequences for *P. vlangalii* using PopART software. Figure S2: Molecular dating for partial species of the genus *Phrynocephalus* based on the concatenated mtDNA. Branch support values are not given for most intra-generic nodes to preserve clarity. C1, C2, and C3 indicate the calibration points placed on nodes, and bars show 95% HPD of divergence date, with the red bar representing the node of *P. vlangalii*. Figure S3: Results of the Mantel test of IBD (A) and IBE (B) analysis for *P. vlangalii* with precise location coordinates. Red lines show the trend of the scatter plot. Figure S4. Response curves for each environmental variable in ENM. Table S1: Information on the sampling localities of *P. vlangalii*, showing locality code and abbreviation, sample size (n), and geographic coordinates for each locality. Table S2: GenBank accession number of each sample and molecular marker. Table S3: mtDNA and nuDNA primers used in this study, and annealing temperatures used in the PCR reactions. Table S4: Sequence information for each gene segment. Table S5: Data on the phylogenetic, molecular dating, and BSP analyses, including partitions, models, and parameters. Table S6: Morphological characters examined in this study. Table S7: Occurrence localities used in ENM. Table S8: Eight bioclimatic factors used in ecological niche modeling. Table S9: Morphological data for each clade. Table S10: Loading and cumulative variation for the principal components. References [74,75,76,77,78,79,80,81,82,83,84,85,86,87,88,89,90,91,92,93,94] are cited in the Supplementary Materials.
