# Supplementary material for: Lineage Diversification and Population Dynamics of the Qinghai Toad-Headed Agama (Phrynocephalus vlangalii) on the Qinghai–Tibet Plateau, with Particular Attention to the Northern Slope of the Kunlun–Arjin Mountains
Source: Animals (Basel). 2025 Jan 31;15(3):400. doi: 10.3390/ani15030400 (PMC11815740; doi:10.3390/ani15030400)
Supplement: Supplementary file 1 [file animals-15-00400-s001.zip › animals-3375003-supplementary 2.pdf]

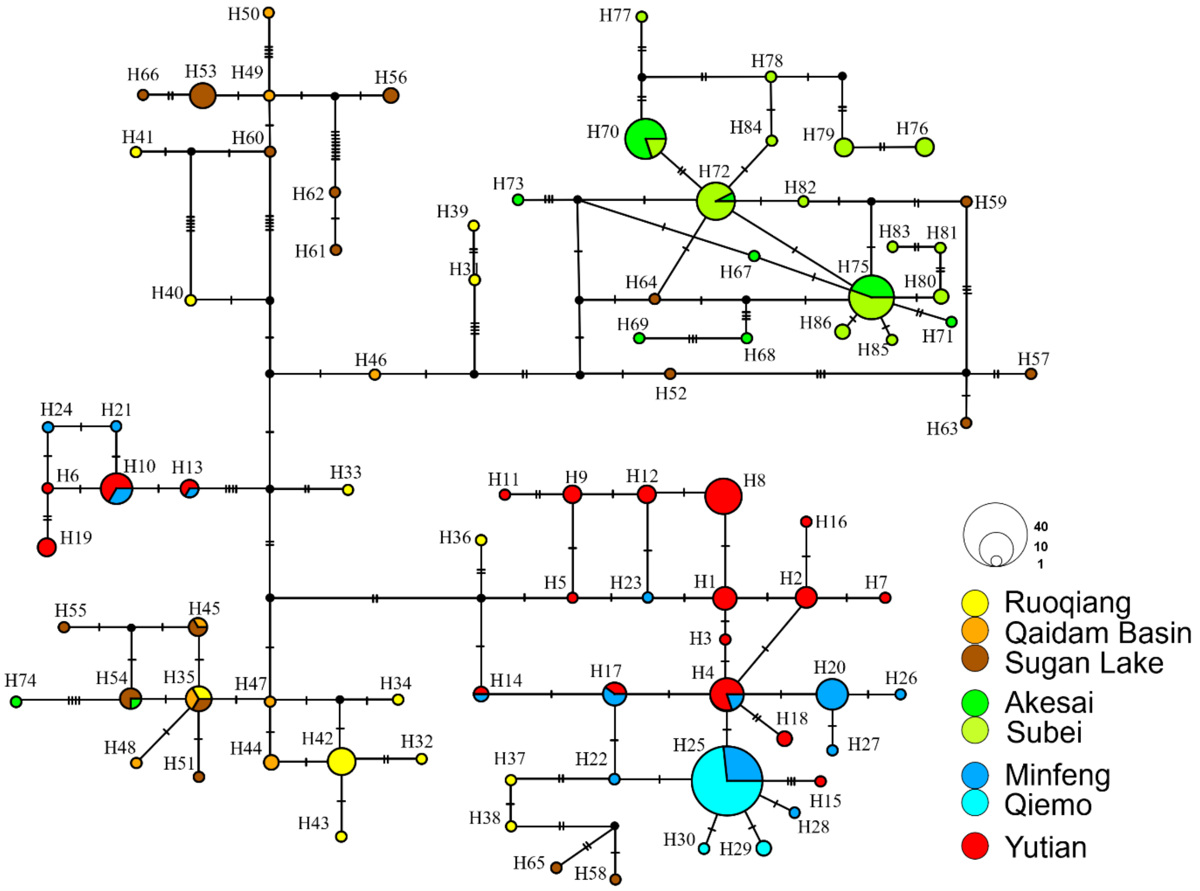

Figure S1. Median-joining network of haplotypes inferred from a total of 130 concatenated nuDNA sequences for *P. vlangalii* using PopART software.

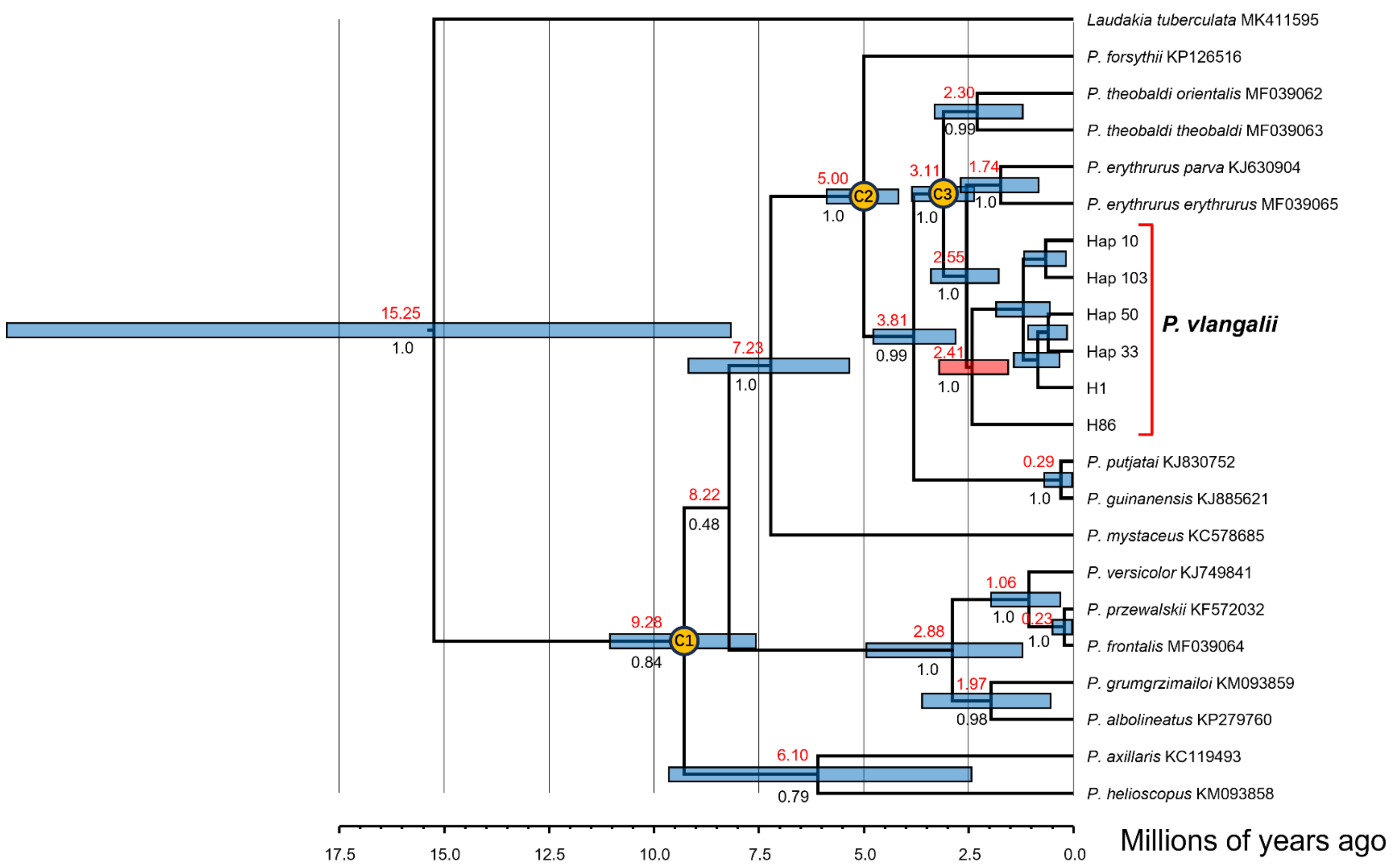

Figure S2. Molecular dating for partial species of the genus *Phrynocephalus* based on the concatenated mtDNA. Branch support values are not given for most intra-generic nodes to preserve clarity. C1, C2 and C3 indicate the calibration points placed on nodes, and bars show 95% HPD of divergence date, the red bar represents the node of *P. vlangalii*.

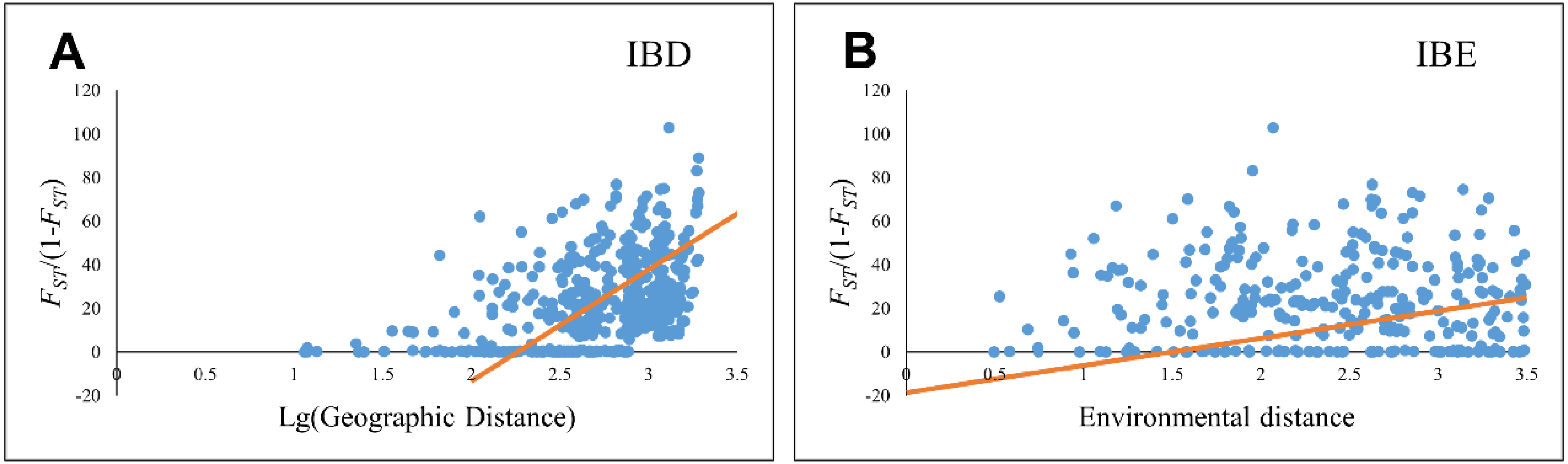

Figure S3. Results of the Mantel test of IBD (A) and IBE (B) analysis for *P. vlangalii* with precise location coordinates. Red lines show the trend of scatter plot.

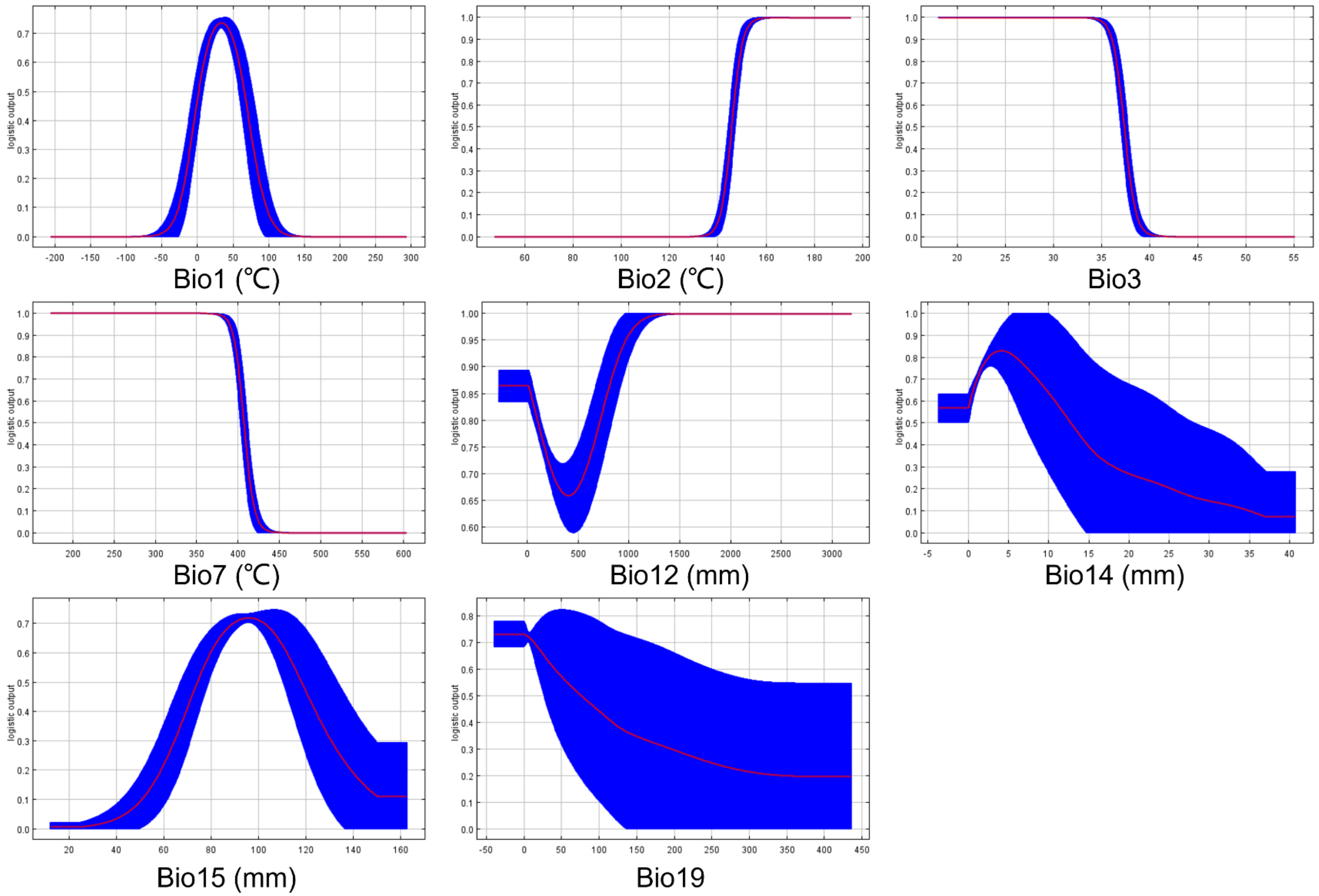

Figure S4. Response curves for each environmental variable in ENM.

Table S1. Information on the sampling localities of *P. vlangalii*, showing locality code and abbreviation, sample size (n) and geographic coordinates for each locality.

Locality code	n	Location	Clade	Longitude (°)	Latitude (°)	Elevation (m)	Reference
1	9	Yutian County, Xinjiang	VI	36.18	81.56	2575	This study
2	3	Yutian County, Xinjiang	VI	36.22	81.68	2835	This study
3	12	Yutian County, Xinjiang	VI	36.31	81.78	3088	This study
4	5	Yutian County, Xinjiang	VI	36.44	81.97	2629	This study
5	8	Minfeng County, Xinjiang	IV	36.67	83.18	2584	This study
6	11	Minfeng County, Xinjiang	IV	37.11	84.53	2391	This study
7	6	Qiemo County, Xinjiang	IV	37.43	85.44	2343	This study
8	12	Qiemo County, Xinjiang	IV	37.33	85.47	2767	This study
9	6	Ruoqiang County, Xinjiang	II	38.43	90.12	3053	This study
10	4	Ruoqiang County, Xinjiang	II	38.92	90.16	3226	This study
11	5	Huatugou Town, Qinghai	II	37.96	91.25	3008	This study
12	14	Sugan Lake, Gansu	II	38.9	93.9	2800	This study
13	14	Akesai County, Gansu	III	39.43	94.26	2539	This study
14	9	Subei County, Gansu	III	39.34	95.32	3068	This study
15	12	Subei County, Gansu	III	39.44	94.97	2489	This study
16	15	Huatugou, Qinghai, China	II	38.23	90.84	2915	[12]
17	4	Mahai, Qinghai, China	II	37.97	94.41	2822	[12]
18	4	Dachaidan, Qinghai, China	II	37.89	95.29	3171	[12]
19	16	Nachitai, Qinghai, China	II	35.88	94.52	3618.3	[12]
20	16	Xidatan, Qinghai, China	II	35.75	94.32	4124	[12]
21	16	Geermu, Qinghai, China	II	36.37	95.03	2808	[12]
22	3	Nuomuhong, Qinghai, China	II	36.52	96.19	2680	[12]
23	16	Delinha, Qinghai, China	II	37.34	97.18	2912	[12]
24	19	Xiangride, Qinghai, China	II	36.02	97.76	3053	[12]
25	16	Dulan, Qinghai, China	II	36.42	98.13	3114	[12]
26	16	Wulan, Qinghai, China	II	36.89	98.53	2913	[12]
27	16	Chaka, Qinghai, China	II	36.65	99.38	3235	[12]
28	16	Gonghe, Qinghai, China	I	36.12	100.44	2877	[12]
29	16	Maduo, Qinghai, China	V	34.9	98.21	4260	[12]
30	16	Maqu, Sichuan, China	V	33.94	102.09	3437	[12]
31	16	Ruoergai, Sichuan, China	V	33.74	102.5	3486	[12]

Table S2. GenBank accession number of each sample and molecular marker.

Species	Voucher Number	Locality code	16S	ND2-tRNA	ND4-tRNA	RAG1	AMEL	Reference
P. vlangalii	GXG1615	1	PQ436688	PQ433912	PQ434042	PQ434171	PQ434301	This study
	GXG1648	1	PQ436689	PQ433913	PQ434043	PQ434172	PQ434302	This study
	GXG1649	1	PQ436690	PQ433914	PQ434044	PQ434173	PQ434303	This study
	GXG1656	1	PQ436691	PQ433915	PQ434045	PQ434174	PQ434304	This study
	GXG1657	1	PQ436692	PQ433916	PQ434046	PQ434175	PQ434305	This study
	GXG1658	1	PQ436693	PQ433917	PQ434047	PQ434176	PQ434306	This study
	GXG1659	1	PQ436694	PQ433918	PQ434048	PQ434177	PQ434307	This study
	GXG1660	1	PQ436695	PQ433919	PQ434049	PQ434178	PQ434308	This study
	GXG1661	1	PQ436696	PQ433920	PQ434050	PQ434179	PQ434309	This study
	Guo9582	2	PQ436737	PQ433961	PQ434091	PQ434220	PQ434350	This study
	Guo9583	2	PQ436738	PQ433962	PQ434092	PQ434221	PQ434351	This study
	Guo9584	2	PQ436739	PQ433963	PQ434093	PQ434222	PQ434352	This study
	Guo9570	3	PQ436725	PQ433949	PQ434079	PQ434208	PQ434338	This study
	Guo9571	3	PQ436726	PQ433950	PQ434080	PQ434209	PQ434339	This study
	Guo9572	3	PQ436727	PQ433951	PQ434081	PQ434210	PQ434340	This study
	Guo9573	3	PQ436728	PQ433952	PQ434082	PQ434211	PQ434341	This study
	Guo9574	3	PQ436729	PQ433953	PQ434083	PQ434212	PQ434342	This study
	Guo9575	3	PQ436730	PQ433954	PQ434084	PQ434213	PQ434343	This study
	Guo9576	3	PQ436731	PQ433955	PQ434085	PQ434214	PQ434344	This study
	Guo9577	3	PQ436732	PQ433956	PQ434086	PQ434215	PQ434345	This study
	Guo9578	3	PQ436733	PQ433957	PQ434087	PQ434216	PQ434346	This study
	Guo9579	3	PQ436734	PQ433958	PQ434088	PQ434217	PQ434347	This study
	Guo9580	3	PQ436735	PQ433959	PQ434089	PQ434218	PQ434348	This study
	Guo9581	3	PQ436736	PQ433960	PQ434090	PQ434219	PQ434349	This study
	Guo9562	4	PQ436720	PQ433944	PQ434074	PQ434203	PQ434333	This study
	Guo9564	4	PQ436721	PQ433945	PQ434075	PQ434204	PQ434334	This study
	Guo9565	4	PQ436722	PQ433946	PQ434076	PQ434205	PQ434335	This study
	Guo9566	4	PQ436723	PQ433947	PQ434077	PQ434206	PQ434336	This study
	Guo9567	4	PQ436724	PQ433948	PQ434078	PQ434207	PQ434337	This study
	GXG1347	5	PQ436674	PQ433898	PQ434028	PQ434157	PQ434287	This study
	GXG1440	5	PQ436681	PQ433905	PQ434035	PQ434164	PQ434294	This study
	GXG1441	5	PQ436682	PQ433906	PQ434036	PQ434165	PQ434295	This study
	GXG1442	5	PQ436683	PQ433907	PQ434037	PQ434166	PQ434296	This study
	GXG1443	5	PQ436684	PQ433908	PQ434038	PQ434167	PQ434297	This study
	GXG1444	5	PQ436685	PQ433909	PQ434039	PQ434168	PQ434298	This study

P. vlangalii	GXG1445	5	PQ436686	PQ433910	PQ434040	PQ434169	PQ434299	This study
	GXG1446	5	PQ436687	PQ433911	PQ434041	PQ434170	PQ434300	This study
	GXG1677	6	PQ436697	PQ433921	PQ434051	PQ434180	PQ434310	This study
	GXG1678	6	PQ436698	PQ433922	PQ434052	PQ434181	PQ434311	This study
	GXG1685	6	PQ436699	PQ433923	PQ434053	PQ434182	PQ434312	This study
	GXG1686	6	PQ436700	PQ433924	PQ434054	PQ434183	PQ434313	This study
	GXG1687	6	PQ436701	PQ433925	PQ434055	PQ434184	PQ434314	This study
	GXG1688	6	PQ436702	PQ433926	PQ434056	PQ434185	PQ434315	This study
	GXG1689	6	PQ436703	PQ433927	PQ434057	PQ434186	PQ434316	This study
	GXG1690	6	PQ436704	PQ433928	PQ434058	PQ434187	PQ434317	This study
	GXG1691	6	PQ436705	PQ433929	PQ434059	PQ434188	PQ434318	This study
	GXG1692	6	PQ436706	PQ433930	PQ434060	PQ434189	PQ434319	This study
	GXG1693	6	PQ436707	PQ433931	PQ434061	PQ434190	PQ434320	This study
	GXG1414	7	PQ436675	PQ433899	PQ434029	PQ434158	PQ434288	This study
	GXG1415	7	PQ436676	PQ433900	PQ434030	PQ434159	PQ434289	This study
	Guo6868	7	PQ436708	PQ433932	PQ434062	PQ434191	PQ434321	This study
	Guo8165	7	PQ436717	PQ433941	PQ434071	PQ434200	PQ434330	This study
	Guo8166	7	PQ436718	PQ433942	PQ434072	PQ434201	PQ434331	This study
	Guo8167	7	PQ436719	PQ433943	PQ434073	PQ434202	PQ434332	This study
	GXG1416	8	PQ436677	PQ433901	PQ434031	PQ434160	PQ434290	This study
	GXG1417	8	PQ436678	PQ433902	PQ434032	PQ434161	PQ434291	This study
	GXG1418	8	PQ436679	PQ433903	PQ434033	PQ434162	PQ434292	This study
	GXG1419	8	PQ436680	PQ433904	PQ434034	PQ434163	PQ434293	This study
	Guo6898	8	PQ436709	PQ433933	PQ434063	PQ434192	PQ434322	This study
	Guo6899	8	PQ436710	PQ433934	PQ434064	PQ434193	PQ434323	This study
	Guo6900	8	PQ436711	PQ433935	PQ434065	PQ434194	PQ434324	This study
	Guo6901	8	PQ436712	PQ433936	PQ434066	PQ434195	PQ434325	This study
	Guo6902	8	PQ436713	PQ433937	PQ434067	PQ434196	PQ434326	This study
	Guo6905	8	PQ436714	PQ433938	PQ434068	PQ434197	PQ434327	This study
	Guo6906	8	PQ436715	PQ433939	PQ434069	PQ434198	PQ434328	This study
	Guo6907	8	PQ436716	PQ433940	PQ434070	PQ434199	PQ434329	This study
	Guo3474	9	PQ436749	PQ433973	PQ434103	PQ434232	PQ434362	This study
	Guo3475	9	PQ436750	PQ433974	PQ434104	PQ434233	PQ434363	This study
	Guo3476	9	PQ436751	PQ433975	PQ434105	PQ434234	PQ434364	This study
	Guo5522	9	PQ436794	PQ434018	PQ434147	PQ434277	PQ434407	This study
	Guo5693	9	PQ436799	PQ434023	PQ434152	PQ434282	PQ434412	This study
	Guo5694	9	PQ436800	PQ434024	PQ434153	PQ434283	PQ434413	This study
	Guo5527	10	PQ436795	PQ434019	PQ434148	PQ434278	PQ434408	This study

P. vlangalii	Guo5718	10	PQ436801	PQ434025	PQ434154	PQ434284	PQ434414	This study
	Guo5719	10	PQ436802	PQ434026	PQ434155	PQ434285	PQ434415	This study
	Guo5720	10	PQ436803	PQ434027	PQ434156	PQ434286	PQ434416	This study
	Guo5496	11	PQ436792	PQ434016	PQ434145	PQ434275	PQ434405	This study
	Guo5497	11	PQ436793	PQ434017	PQ434146	PQ434276	PQ434406	This study
	Guo5670	11	PQ436796	PQ434020	PQ434149	PQ434279	PQ434409	This study
	Guo5671	11	PQ436797	PQ434021	PQ434150	PQ434280	PQ434410	This study
	Guo5672	11	PQ436798	PQ434022	PQ434151	PQ434281	PQ434411	This study
	Guo3425	12	PQ436744	PQ433968	PQ434098	PQ434227	PQ434357	This study
	Guo3426	12	PQ436745	PQ433969	PQ434099	PQ434228	PQ434358	This study
	Guo3427	12	PQ436746	PQ433970	PQ434100	PQ434229	PQ434359	This study
	Guo3428	12	PQ436747	PQ433971	PQ434101	PQ434230	PQ434360	This study
	Guo3429	12	PQ436748	PQ433972	PQ434102	PQ434231	PQ434361	This study
	Guo4085	12	PQ436752	PQ433976	PQ434106	PQ434235	PQ434365	This study
	Guo4699	12	PQ436753	PQ433977	PQ434107	PQ434236	PQ434366	This study
	Guo4700	12	PQ436754	PQ433978	PQ434108	PQ434237	PQ434367	This study
	Guo4701	12	PQ436755	PQ433979	PQ434109	PQ434238	PQ434368	This study
	Guo4702	12	PQ436756	PQ433980	PQ434110	PQ434239	PQ434369	This study
	Guo4703	12	PQ436757	PQ433981	PQ434111	PQ434240	PQ434370	This study
	Guo4704	12	PQ436758	PQ433982	PQ434112	PQ434241	PQ434371	This study
	Guo4705	12	PQ436759	PQ433983	PQ434113	PQ434242	PQ434372	This study
	Guo4706	12	PQ436760	PQ433984	PQ434114	PQ434243	PQ434373	This study
	Guo2580	13	PQ436740	PQ433964	PQ434094	PQ434223	PQ434353	This study
	Guo2602	13	PQ436741	PQ433965	PQ434095	PQ434224	PQ434354	This study
	Guo2603	13	PQ436742	PQ433966	PQ434096	PQ434225	PQ434355	This study
	Guo3383	13	PQ436743	PQ433967	PQ434097	PQ434226	PQ434356	This study
	Guo4966	13	PQ436761	PQ433985	PQ434115	PQ434244	PQ434374	This study
	Guo4967	13	PQ436762	PQ433986	PQ434116	PQ434245	PQ434375	This study
	Guo4968	13	PQ436763	PQ433987	PQ434117	PQ434246	PQ434376	This study
	Guo4969	13	PQ436764	PQ433988	PQ434118	PQ434247	PQ434377	This study
	Guo4970	13	PQ436765	PQ433989	PQ434119	PQ434248	PQ434378	This study
	Guo5078	13	PQ436775	PQ433999	PQ434129	PQ434258	PQ434388	This study
	Guo5111	13	PQ436776	PQ434000	PQ434130	PQ434259	PQ434389	This study
	Guo5112	13	PQ436777	PQ434001	PQ434131	PQ434260	PQ434390	This study
	Guo5113	13	PQ436778	PQ434002	PQ434132	PQ434261	PQ434391	This study
	Guo5114	13	PQ436779	PQ434003	PQ434133	PQ434262	PQ434392	This study
	Guo4989	14	PQ436766	PQ433990	PQ434120	PQ434249	PQ434379	This study
	Guo4990	14	PQ436767	PQ433991	PQ434121	PQ434250	PQ434380	This study

P. vlangalii	Guo4991	14	PQ436768	PQ433992	PQ434122	PQ434251	PQ434381	This study
	Guo4992	14	PQ436769	PQ433993	PQ434123	PQ434252	PQ434382	This study
	Guo4993	14	PQ436770	PQ433994	PQ434124	PQ434253	PQ434383	This study
	Guo4994	14	PQ436771	PQ433995	PQ434125	PQ434254	PQ434384	This study
	Guo4995	14	PQ436772	PQ433996	PQ434126	PQ434255	PQ434385	This study
	Guo4996	14	PQ436773	PQ433997	PQ434127	PQ434256	PQ434386	This study
	Guo4997	14	PQ436774	PQ433998	PQ434128	PQ434257	PQ434387	This study
	Guo5188	15	PQ436780	PQ434004	PQ434134	PQ434263	PQ434393	This study
	Guo5189	15	PQ436781	PQ434005	PQ434135	PQ434264	PQ434394	This study
	Guo5190	15	PQ436782	PQ434006	PQ434136	PQ434265	PQ434395	This study
	Guo5191	15	PQ436783	PQ434007	PQ434137	PQ434266	PQ434396	This study
	Guo5192	15	PQ436784	PQ434008	PQ434138	PQ434267	PQ434397	This study
	Guo5193	15	PQ436785	PQ434009	PQ434139	PQ434268	PQ434398	This study
	Guo5194	15	PQ436786	PQ434010	PQ434140	PQ434269	PQ434399	This study
	Guo5195	15	PQ436787	PQ434011	PQ434141	PQ434270	PQ434400	This study
	Guo5196	15	PQ436788	PQ434012	PQ434142	PQ434271	PQ434401	This study
	Guo5226	15	PQ436789	PQ434013	—	PQ434272	PQ434402	This study
	Guo5265	15	PQ436790	PQ434014	PQ434143	PQ434273	PQ434403	This study
	Guo5276	15	PQ436791	PQ434015	PQ434144	PQ434274	PQ434404	This study
	SGH_F002	12	MT974885	MW005317	MW005851	MT989757	—	[12]
	SGH_F003	12	MT974886	MW005318	MW005852	MT989758	—	[12]
	SGH_F004	12	MT974887	MW005319	MW005853	MT989759	—	[12]
	SGH_F006	12	MT974888	MW005320	MW005854	MT989760	—	[12]
	SGH_F008	12	MT974889	MW005321	MW005855	MT989761	—	[12]
	SGH_F011	12	MT974890	MW005322	MW005856	MT989762	—	[12]
	SGH_F012	12	MT974891	MW005323	MW005857	MT989763	—	[12]
	SGH_F014	12	MT974892	MW005324	MW005858	MT989764	—	[12]
	SGH_M002	12	MT974893	MW005325	MW005859	MT989765	—	[12]
	SGH_M003	12	MT974894	MW005326	MW005860	MT989766	—	[12]
	SGH_M004	12	MT974895	MW005327	MW005861	MT989767	—	[12]
	SGH_M005	12	MT974896	MW005328	MW005862	MT989768	—	[12]
	SGH_M006	12	MT974897	MW005329	MW005863	MT989769	—	[12]
	SGH_M008	12	MT974898	MW005330	MW005864	MT989770	—	[12]
	SGH_M013	12	MT974899	MW005331	MW005865	MT989771	—	[12]
	SGH_M014	12	MT974900	MW005332	MW005866	MT989772	—	[12]
	AKS_F005	13	MT974694	MW004913	MW005447	MT989566	—	[12]
	AKS_F007	13	MT974695	MW004914	MW005448	MT989567	—	[12]
	AKS_F009	13	MT974696	MW004915	MW005449	MT989568	—	[12]

P. vlangalii	AKS_F010	13	MT974697	MW004916	MW005450	MT989569	—	[12]	
	AKS_F011	13	MT974698	MW004917	MW005451	MT989570	—	[12]	
	AKS_F012	13	MT974699	MW004918	MW005452	MT989571	—	[12]	
	AKS_F015	13	MT974700	MW004919	MW005453	MT989572	—	[12]	
	AKS_F019	13	MT974701	MW004920	MW005454	MT989573	—	[12]	
	AKS_M001	13	MT974702	MW004921	MW005455	MT989574	—	[12]	
	AKS_M003	13	MT974703	MW004922	MW005456	MT989575	—	[12]	
	AKS_M004	13	MT974704	MW004923	MW005457	MT989576	—	[12]	
	AKS_M009	13	MT974705	MW004924	MW005458	MT989577	—	[12]	
	AKS_M012	13	MT974706	MW004925	MW005459	MT989578	—	[12]	
	AKS_M015	13	MT974707	MW004926	MW005460	MT989579	—	[12]	
	AKS_M016	13	MT974708	MW004927	MW005461	MT989580	—	[12]	
	AKS_M020	13	MT974709	MW004928	MW005462	MT989581	—	[12]	
	HTG_F006	16	MT974806	MW005155	MW005689	MT989678	—	[12]	
	HTG_F012	16	MT974807	MW005156	MW005690	MT989679	—	[12]	
	HTG_F013	16	MT974808	MW005157	MW005691	MT989680	—	[12]	
	HTG_F014	16	MT974809	MW005158	MW005692	MT989681	—	[12]	
	HTG_F019	16	MT974810	MW005159	MW005693	MT989682	—	[12]	
	HTG_F020	16	MT974811	MW005160	MW005694	MT989683	—	[12]	
	HTG_F021	16	MT974812	MW005161	MW005695	MT989684	—	[12]	
	HTG_M001	16	MT974813	MW005162	MW005696	MT989685	—	[12]	
	HTG_M003	16	MT974814	MW005163	MW005697	MT989686	—	[12]	
	HTG_M005	16	MT974815	MW005164	MW005698	MT989687	—	[12]	
	HTG_M006	16	MT974816	MW005165	MW005699	MT989688	—	[12]	
	HTG_M012	16	MT974817	MW005166	MW005700	MT989689	—	[12]	
	HTG_M013	16	MT974818	MW005167	MW005701	MT989690	—	[12]	
	HTG_M014	16	MT974819	MW005168	MW005702	MT989691	—	[12]	
	HTG_M016	16	MT974820	MW005169	MW005703	MT989692	—	[12]	
	MH_F001	17	MT974937	MW005230	MW005764	MT989809	—	[12]	
	MH_F002	17	MT974938	MW005231	MW005765	MT989810	—	[12]	
	MH_F004	17	MT974939	MW005232	MW005766	MT989811	—	[12]	
	MH_M003	17	MT974940	MW005233	MW005767	MT989812	—	[12]	
	DCD_F001	18	MT974933	MW004945	MW005479	MT989805	—	[12]	
	DCD_F004	18	MT974934	MW004946	MW005480	MT989806	—	[12]	
	DCD_M001	18	MT974935	MW004947	MW005481	MT989807	—	[12]	
	DCD_M003	18	MT974936	MW004948	MW005482	MT989808	—	[12]	
	NCT_F006	19	MT974853	MW005266	MW005800	MT989725	—	[12]	
	NCT_F008	19	MT974854	MW005267	MW005801	MT989726	—	[12]	

P. vlangalii	NCT_F009	19	MT974855	MW005268	MW005802	MT989727	—	[12]
	NCT_F010	19	MT974856	MW005269	MW005803	MT989728	—	[12]
	NCT_F012	19	MT974857	MW005270	MW005804	MT989729	—	[12]
	NCT_F015	19	MT974858	MW005271	MW005805	MT989730	—	[12]
	NCT_F018	19	MT974859	MW005272	MW005806	MT989731	—	[12]
	NCT_F019	19	MT974860	MW005273	MW005807	MT989732	—	[12]
	NCT_M005	19	MT974861	MW005274	MW005808	MT989733	—	[12]
	NCT_M006	19	MT974862	MW005275	MW005809	MT989734	—	[12]
	NCT_M007	19	MT974863	MW005276	MW005810	MT989735	—	[12]
	NCT_M009	19	MT974864	MW005277	MW005811	MT989736	—	[12]
	NCT_M010	19	MT974865	MW005278	MW005812	MT989737	—	[12]
	NCT_M014	19	MT974866	MW005279	MW005813	MT989738	—	[12]
	NCT_M015	19	MT974867	MW005280	MW005814	MT989739	—	[12]
	NCT_M026	19	MT974868	MW005281	MW005815	MT989740	—	[12]
	XDT_F002	20	MT974917	MW005402	MW005936	MT989789	—	[12]
	XDT_F006	20	MT974918	MW005403	MW005937	MT989790	—	[12]
	XDT_F007	20	MT974919	MW005404	MW005938	MT989791	—	[12]
	XDT_F009	20	MT974920	MW005405	MW005939	MT989792	—	[12]
	XDT_F010	20	MT974921	MW005406	MW005940	MT989793	—	[12]
	XDT_F014	20	MT974922	MW005407	MW005941	MT989794	—	[12]
	XDT_F015	20	MT974923	MW005408	MW005942	MT989795	—	[12]
	XDT_F024	20	MT974924	MW005409	MW005943	MT989796	—	[12]
	XDT_M002	20	MT974925	MW005410	MW005944	MT989797	—	[12]
	XDT_M006	20	MT974926	MW005411	MW005945	MT989798	—	[12]
	XDT_M008	20	MT974927	MW005412	MW005946	MT989799	—	[12]
	XDT_M010	20	MT974928	MW005413	MW005947	MT989800	—	[12]
	XDT_M011	20	MT974929	MW005414	MW005948	MT989801	—	[12]
	XDT_M013	20	MT974930	MW005415	MW005949	MT989802	—	[12]
	XDT_M014	20	MT974931	MW005416	MW005950	MT989803	—	[12]
	XDT_M015	20	MT974932	MW005417	MW005951	MT989804	—	[12]
	GEM_F001	21	MT974758	MW005045	MW005579	MT989630	—	[12]
	GEM_F003	21	MT974759	MW005046	MW005580	MT989631	—	[12]
	GEM_F007	21	MT974760	MW005047	MW005581	MT989632	—	[12]
	GEM_F009	21	MT974761	MW005048	MW005582	MT989633	—	[12]
	GEM_F010	21	MT974762	MW005049	MW005583	MT989634	—	[12]
	GEM_F011	21	MT974763	MW005050	MW005584	MT989635	—	[12]
	GEM_F015	21	MT974764	MW005051	MW005585	MT989636	—	[12]
	GEM_F017	21	MT974765	MW005052	MW005586	MT989637	—	[12]

P. vlangalii	GEM_M001	21	MT974766	MW005053	MW005587	MT989638	—	[12]
	GEM_M002	21	MT974767	MW005054	MW005588	MT989639	—	[12]
	GEM_M003	21	MT974768	MW005055	MW005589	MT989640	—	[12]
	GEM_M010	21	MT974769	MW005056	MW005590	MT989641	—	[12]
	GEM_M015	21	MT974770	MW005057	MW005591	MT989642	—	[12]
	GEM_M016	21	MT974771	MW005058	MW005592	MT989643	—	[12]
	GEM_M020	21	MT974772	MW005059	MW005593	MT989644	—	[12]
	GEM_M023	21	MT974773	MW005060	MW005594	MT989645	—	[12]
	NMH_M001	22	MT974941	MW005282	MW005816	MT989813	—	[12]
	NMH_M002	22	MT974942	MW005283	MW005817	MT989814	—	[12]
	NMH_M003	22	MT974943	MW005284	MW005818	MT989815	—	[12]
	DLH_F001	23	MT974742	MW004949	MW005483	MT989614	—	[12]
	DLH_F002	23	MT974743	MW004950	MW005484	MT989615	—	[12]
	DLH_F003	23	MT974744	MW004951	MW005485	MT989616	—	[12]
	DLH_F010	23	MT974745	MW004952	MW005486	MT989617	—	[12]
	DLH_F011	23	MT974746	MW004953	MW005487	MT989618	—	[12]
	DLH_F014	23	MT974747	MW004954	MW005488	MT989619	—	[12]
	DLH_F017	23	MT974748	MW004955	MW005489	MT989620	—	[12]
	DLH_F019	23	MT974749	MW004956	MW005490	MT989621	—	[12]
	DLH_M002	23	MT974750	MW004957	MW005491	MT989622	—	[12]
	DLH_M007	23	MT974751	MW004958	MW005492	MT989623	—	[12]
	DLH_M008	23	MT974752	MW004959	MW005493	MT989624	—	[12]
	DLH_M009	23	MT974753	MW004960	MW005494	MT989625	—	[12]
	DLH_M013	23	MT974754	MW004961	MW005495	MT989626	—	[12]
	DLH_M015	23	MT974755	MW004962	MW005496	MT989627	—	[12]
	DLH_M016	23	MT974756	MW004963	MW005497	MT989628	—	[12]
	DLH_M019	23	MT974757	MW004964	MW005498	MT989629	—	[12]
	XRD_F001	24	MT974954	MW005428	MW005962	MT989826	—	[12]
	XRD_F002	24	MT974955	MW005429	MW005963	MT989827	—	[12]
	XRD_F003	24	MT974956	MW005430	MW005964	MT989828	—	[12]
	XRD_F004	24	MT974957	MW005431	MW005965	MT989829	—	[12]
	XRD_F005	24	MT974958	MW005432	MW005966	MT989830	—	[12]
	XRD_F006	24	MT974959	MW005433	MW005967	MT989831	—	[12]
	XRD_F007	24	MT974960	MW005434	MW005968	MT989832	—	[12]
	XRD_F008	24	MT974961	MW005435	MW005969	MT989833	—	[12]
	XRD_F011	24	MT974962	MW005436	MW005970	MT989834	—	[12]
	XRD_F012	24	MT974963	MW005437	MW005971	MT989835	—	[12]
	XRD_F013	24	MT974964	MW005438	MW005972	MT989836	—	[12]

XRD_M001	24	MT974965	MW005439	MW005973	MT989837	—	[12]
XRD_M002	24	MT974966	MW005440	MW005974	MT989838	—	[12]
XRD_M202	24	MT975178	MW005441	MW005975	MT990047	—	[12]
XRD_M205	24	MT975179	MW005442	MW005976	MT990048	—	[12]
XRD_M206	24	MT975180	MW005443	MW005977	MT990049	—	[12]
XRD_M208	24	MT975181	MW005444	MW005978	MT990050	—	[12]
XRD_M209	24	MT975182	MW005445	MW005979	MT990051	—	[12]
XRD_M213	24	MT975183	MW005446	MW005980	MT990052	—	[12]
DL_F101	25	MT974726	MW004965	MW005499	MT989598	—	[12]
DL_F102	25	MT974727	MW004966	MW005500	MT989599	—	[12]
DL_F103	25	MT974728	MW004967	MW005501	MT989600	—	[12]
DL_F104	25	MT974729	MW004968	MW005502	MT989601	—	[12]
DL_F105	25	MT974730	MW004969	MW005503	MT989602	—	[12]
DL_F106	25	MT974731	MW004970	MW005504	MT989603	—	[12]
DL_F107	25	MT974732	MW004971	MW005505	MT989604	—	[12]
DL_F111	25	MT974733	MW004972	MW005506	MT989605	—	[12]
DL_M102	25	MT974734	MW004973	MW005507	MT989606	—	[12]
DL_M103	25	MT974735	MW004974	MW005508	MT989607	—	[12]
DL_M104	25	MT974736	MW004975	MW005509	MT989608	—	[12]
DL_M105	25	MT974737	MW004976	MW005510	MT989609	—	[12]
DL_M106	25	MT974738	MW004977	MW005511	MT989610	—	[12]
DL_M107	25	MT974739	MW004978	MW005512	MT989611	—	[12]
DL_M108	25	MT974740	MW004979	MW005513	MT989612	—	[12]
DL_M109	25	MT974741	MW004980	MW005514	MT989613	—	[12]
WL_F001	26	MT974901	MW005370	MW005904	MT989773	—	[12]
WL_F006	26	MT974902	MW005371	MW005905	MT989774	—	[12]
WL_F007	26	MT974903	MW005372	MW005906	MT989775	—	[12]
WL_F009	26	MT974904	MW005373	MW005907	MT989776	—	[12]
WL_F010	26	MT974905	MW005374	MW005908	MT989777	—	[12]
WL_F016	26	MT974906	MW005375	MW005909	MT989778	—	[12]
WL_F017	26	MT974907	MW005376	MW005910	MT989779	—	[12]
WL_F018	26	MT974908	MW005377	MW005911	MT989780	—	[12]
WL_M008	26	MT974909	MW005378	MW005912	MT989781	—	[12]
WL_M010	26	MT974910	MW005379	MW005913	MT989782	—	[12]
WL_M016	26	MT974911	MW005380	MW005914	MT989783	—	[12]
WL_M017	26	MT974912	MW005381	MW005915	MT989784	—	[12]
WL_M018	26	MT974913	MW005382	MW005916	MT989785	—	[12]
WL_M019	26	MT974914	MW005383	MW005917	MT989786	—	[12]

P. vlangalii	WL_M020	26	MT974915	MW005384	MW005918	MT989787	—	[12]
	WL_M022	26	MT974916	MW005385	MW005919	MT989788	—	[12]
	CK_F001	27	MT974710	MW004929	MW005463	MT989582	—	[12]
	CK_F004	27	MT974711	MW004930	MW005464	MT989583	—	[12]
	CK_F015	27	MT974712	MW004931	MW005465	MT989584	—	[12]
	CK_F016	27	MT974713	MW004932	MW005466	MT989585	—	[12]
	CK_F018	27	MT974714	MW004933	MW005467	MT989586	—	[12]
	CK_F019	27	MT974715	MW004934	MW005468	MT989587	—	[12]
	CK_F020	27	MT974716	MW004935	MW005469	MT989588	—	[12]
	CK_F021	27	MT974717	MW004936	MW005470	MT989589	—	[12]
	CK_M001	27	MT974718	MW004937	MW005471	MT989590	—	[12]
	CK_M002	27	MT974719	MW004938	MW005472	MT989591	—	[12]
	CK_M010	27	MT974720	MW004939	MW005473	MT989592	—	[12]
	CK_M012	27	MT974721	MW004940	MW005474	MT989593	—	[12]
	CK_M013	27	MT974722	MW004941	MW005475	MT989594	—	[12]
	CK_M017	27	MT974723	MW004942	MW005476	MT989595	—	[12]
	CK_M018	27	MT974724	MW004943	MW005477	MT989596	—	[12]
	CK_M020	27	MT974725	MW004944	MW005478	MT989597	—	[12]
	GH1_F001	28	MT974774	MW005061	MW005595	MT989646	—	[12]
	GH1_F008	28	MT974775	MW005062	MW005596	MT989647	—	[12]
	GH1_F010	28	MT974776	MW005063	MW005597	MT989648	—	[12]
	GH1_F011	28	MT974777	MW005064	MW005598	MT989649	—	[12]
	GH1_F012	28	MT974778	MW005065	MW005599	MT989650	—	[12]
	GH1_F013	28	MT974779	MW005066	MW005600	MT989651	—	[12]
	GH1_F014	28	MT974780	MW005067	MW005601	MT989652	—	[12]
	GH1_F020	28	MT974781	MW005068	MW005602	MT989653	—	[12]
	GH1_M002	28	MT974782	MW005069	MW005603	MT989654	—	[12]
	GH1_M003	28	MT974783	MW005070	MW005604	MT989655	—	[12]
	GH1_M006	28	MT974784	MW005071	MW005605	MT989656	—	[12]
	GH1_M009	28	MT974785	MW005072	MW005606	MT989657	—	[12]
	GH1_M010	28	MT974786	MW005073	MW005607	MT989658	—	[12]
	GH1_M011	28	MT974787	MW005074	MW005608	MT989659	—	[12]
	GH1_M013	28	MT974788	MW005075	MW005609	MT989660	—	[12]
	GH1_M015	28	MT974789	MW005076	MW005610	MT989661	—	[12]
	MD_F001	29	MT974821	MW005198	MW005732	MT989693	—	[12]
	MD_F005	29	MT974822	MW005199	MW005733	MT989694	—	[12]
	MD_F006	29	MT974823	MW005200	MW005734	MT989695	—	[12]
	MD_F008	29	MT974824	MW005201	MW005735	MT989696	—	[12]

P. vlangalii	MD_F009	29	MT974825	MW005202	MW005736	MT989697	—	[12]
	MD_F017	29	MT974826	MW005203	MW005737	MT989698	—	[12]
	MD_F019	29	MT974827	MW005204	MW005738	MT989699	—	[12]
	MD_F020	29	MT974828	MW005205	MW005739	MT989700	—	[12]
	MD_M001	29	MT974829	MW005206	MW005740	MT989701	—	[12]
	MD_M003	29	MT974830	MW005207	MW005741	MT989702	—	[12]
	MD_M005	29	MT974831	MW005208	MW005742	MT989703	—	[12]
	MD_M006	29	MT974832	MW005209	MW005743	MT989704	—	[12]
	MD_M008	29	MT974833	MW005210	MW005744	MT989705	—	[12]
	MD_M009	29	MT974834	MW005211	MW005745	MT989706	—	[12]
	MD_M013	29	MT974835	MW005212	MW005746	MT989707	—	[12]
	MD_M014	29	MT974836	MW005213	MW005747	MT989708	—	[12]
	MQ_F006	30	MT974837	MW005250	MW005784	MT989709	—	[12]
	MQ_F008	30	MT974838	MW005251	MW005785	MT989710	—	[12]
	MQ_F011	30	MT974839	MW005252	MW005786	MT989711	—	[12]
	MQ_F012	30	MT974840	MW005253	MW005787	MT989712	—	[12]
	MQ_F017	30	MT974841	MW005254	MW005788	MT989713	—	[12]
	MQ_F021	30	MT974842	MW005255	MW005789	MT989714	—	[12]
	MQ_F023	30	MT974843	MW005256	MW005790	MT989715	—	[12]
	MQ_F024	30	MT974844	MW005257	MW005791	MT989716	—	[12]
	MQ_M005	30	MT974845	MW005258	MW005792	MT989717	—	[12]
	MQ_M011	30	MT974846	MW005259	MW005793	MT989718	—	[12]
	MQ_M012	30	MT974847	MW005260	MW005794	MT989719	—	[12]
	MQ_M013	30	MT974848	MW005261	MW005795	MT989720	—	[12]
	MQ_M014	30	MT974849	MW005262	MW005796	MT989721	—	[12]
	MQ_M015	30	MT974850	MW005263	MW005797	MT989722	—	[12]
	MQ_M016	30	MT974851	MW005264	MW005798	MT989723	—	[12]
	MQ_M017	30	MT974852	MW005265	MW005799	MT989724	—	[12]
	REG_F002	31	MT974869	MW005285	MW005819	MT989741	—	[12]
	REG_F006	31	MT974870	MW005286	MW005820	MT989742	—	[12]
	REG_F010	31	MT974871	MW005287	MW005821	MT989743	—	[12]
	REG_F015	31	MT974872	MW005288	MW005822	MT989744	—	[12]
	REG_F020	31	MT974873	MW005289	MW005823	MT989745	—	[12]
	REG_F024	31	MT974874	MW005290	MW005824	MT989746	—	[12]
	REG_F025	31	MT974875	MW005291	MW005825	MT989747	—	[12]
	REG_F026	31	MT974876	MW005292	MW005826	MT989748	—	[12]
	REG_M003	31	MT974877	MW005293	MW005827	MT989749	—	[12]
	REG_M007	31	MT974878	MW005294	MW005828	MT989750	—	[12]

P. vlangalii	REG_M008	31	MT974879	MW005295	MW005829	MT989751	—	[12]
	REG_M011	31	MT974880	MW005296	MW005830	MT989752	—	[12]
	REG_M013	31	MT974881	MW005297	MW005831	MT989753	—	[12]
	REG_M014	31	MT974882	MW005298	MW005832	MT989754	—	[12]
	REG_M016	31	MT974883	MW005299	MW005833	MT989755	—	[12]
	REG_M022	31	MT974884	MW005300	MW005834	MT989756	—	[12]
P. przewalskii	—	—	MT975176	MW005137	MW005671	MT990097	KC551360	[12,13]
P. putjatai	—	—	MT975047	MW005354	MW005888	MT989919	KC551370	[12,13]
	—	—	KJ830752	KJ830752	KJ830752	—	—	[77]
P. erythrurus	—	—	KJ630904	KJ630904	KJ630904	—	—	[78]
	—	—	MF039065	MF039065	MF039065	—	—	[79]
P. theobaldi	—	—	MF039062	MF039062	MF039062	—	—	[79]
	—	—	MF039063	MF039063	MF039063	—	—	[79]
P. axillaris	—	—	KC119493	KC119493	KC119493	—	—	[80]
P. forsythii	—	—	KP126516	KP126516	KP126516	—	—	[81]
P. guinanensis	—	—	KJ885621	KJ885621	KJ885621	—	—	[82]
P. przewalskii	—	—	KF572032	KF572032	KF572032	—	—	[83]
P. frontalis	—	—	MF039064	MF039064	MF039064	—	—	[79]
P. helioscopus	—	—	KM093858	KM093858	KM093858	—	—	[84]
P. mystaceus	—	—	KC578685	KC578685	KC578685	—	—	[85]
P. versicolor	—	—	KJ749841	KJ749841	KJ749841	—	—	[86]
P. grumgrzimailoi	—	—	KM093859	KM093859	KM093859	—	—	[87]
P. albolineatus	—	—	KP279760	KP279760	KP279760	—	—	[88]
Laudakia tuberculata	—	—	MK411595	MK411595	MK411595	—	—	[89]

Table S3. mtDNA and nuDNA primers used in this study, and annealing temperatures used in the PCR reactions.

Sequence names	Primer name	Primer sequence	Annealing temperature (°C)	Reference
16S	L02510	5'-CGC CTG TTT ATC AAA AAC AT-3'	57.8	[74]
	H03063	5'-CTC CGG TTT GAA CTC AGA TC-3'		
ND2 and adjacent tRNAs	L5002	5'-AAC CAA MCA CAA CTR CGA AAA AC-3'	54.9	[28]
	H5617b	5'-AAA ATA TCT GAG TTG CAT TCA G-3'		
ND4 and adjacent tRNAs	ND4-F	5'-TGA CTA CCA AAA GCT CAT GTA GAA GC-3'	52.8	[75]
	ND4-Leu-R	5'-CAT TAC TTT TAC TTG GAT TTG CAC CA-3'		
RAG-1	Rag-1-7s	5'-TCA RGC AAA CCT TCA GAA CC-3'	65.0	[14]
	Rag-1-7a	5'-CAG GAA CAR AGT TAG GCA CA-3'		
AMEL	LAM2N	5′-TATCCACGTTATGGCTATGAACC-3′	50.0	[76]
	HAM	5′-CACTTCYTCYTKCTTGGTYT-3′		

Table S4. Sequence information of each gene segment.

Sequence names	Length (bp)	Base content	C	PI	V
16S	480	T(23.0%)，C(22.6%)，A(35.2%)，G(19.2%)	458	17	22
ND2	468	T(25.6%)，C(28.7%)，A(39.8%)，G(5.9%)	383	64	85
adjacent tRNAs of ND2	105	T(24.5%)，C(23.7%)，A(35.1%)，G(16.7%)	89	13	16
ND4	660	T(25.4%)，C(26.1%)，A(36.9%)，G(11.6%)	571	72	89
adjacent tRNAs of ND4	143	T(29.0%)，C(22.4%)，A(33.8%)，G(14.8%)	137	5	6
AMEL	380	T(17.5%)，C(35.4%)，A(29.1%)，G(18.0%)	367	5	13
RAG-1	897	T(24.1%)，C(22.8%)，A(29.6%)，G(23.5%)	849	33	48

Table S5. Data on the phylogenetic, molecular dating and BSP analyses, including partitions, models and parameters.

Analysis	Software	Dataset	Model	Partition identity	Length	Clock model	Tree model(linked)	Run Specifications
Phylogenetic trees Bayesian Inference for mtDNA	MrBayes	All sequences of *P. vlangalii* and two outgroups	GTR+I+G	16S, tRNA (ND4), RAG1, AMEL	1900			2 runs; 2×10^{7} generations; 1×10^{3} sampling frequency; 25% burn-in
			HKY+I+G	ND2-1st, tRNA (ND2), ND4-1st	481			
			HKY+I	ND2-2nd, ND4-2nd	376			
			HKY+G	ND2-3rd, ND4-3rd	376			
Phylogenetic trees Maximum Likelihood for mtDNA	IQ-TREE	All sequences of *P. vlangalii* and two outgroups	TRN+I+G	16S, AMEL	860			Ultrafast-bootstrap (UFBoot) x5000
			HKY+G	ND2-1st	156			
			TRN+I	ND2-2nd	156			
			TRN+G	ND2-3rd, ND4-3rd	376			
			GTR+G	ND4-1st, tRNA (ND2, ND4)	468			
			HKY+I	ND4-2nd	220			
			GTR+I+G	RAG1	897			
			HKY+I	ND2-2nd, ND4-2nd	376			
			HKY+G	ND2-3rd, ND4-3rd	376			
Molecular dating with calibration points	BEAST	6 randomly selected sequences from each clade in *P. vlangalii* and 16 outgroups	GTR+I+G	16S	486	Relaxed Uncorrelated Lognormal	Birth-Death Process model	2 runs; 4×10^{7} generations; 2×10^{3} sampling frequency; 25% burn-in
			GTR+G	ND2-1st	156			
			TRN+G	ND2-2nd, ND2-3rd, ND4-3rd	534			
			TRN+I+G	ND4-1st, tRNA (ND2, ND4)	482			
			HKY+G	ND4-2nd	222			
Molecular dating within *P. vlangalii*	BEAST	All sequences in *P. vlangalii*	HKY+I+G	16S, tRNA (ND2+ND4), ND2-1st, ND4-1st	1104	Relaxed Uncorrelated Lognormal	Constant-size Coalescence Random starting tree	2 runs; 3×10^{8} generations; 5×10^{3} sampling frequency; 25% burn-in
			HKY+I	ND2-2nd, ND4-2nd	376			
			HKY+G	ND2-3rd, ND4-3rd	376			
BSP analysis	BEAST	All sequences in *P. vlangalii* Clade I	TRN	16S, tRNA (ND2+ND4), ND4-1st	948	Strict clock	Coalescent: Bayesian Skyline Plot	2 runs; 1×10^{7} generations; 1×10^{3} sampling frequency; 25% burn-in
			HKY	ND2-1st, ND2-2nd, ND2-3rd, ND4-2nd, ND4-3rd	908			
BSP analysis	BEAST	All sequences in *P. vlangalii* Clade II	TRN	16S, ND2-1st, tRNA (ND2+ND4), ND4-1st	1104	Strict clock	Coalescent: Bayesian Skyline Plot	2 runs; 2×10^{8} generations; 2×10^{3} sampling frequency; 25% burn-in
			HKY	ND2-2nd, ND4-2nd, ND2-3rd, ND4-3rd	752			
BSP analysis	BEAST	All sequences in *P. vlangalii* Clade III	HKY	16S, tRNA (ND2+ND4), ND2-2nd, ND2-3rd, ND4-1st, ND4-2nd, ND4-3rd	1700	Strict clock	Coalescent: Bayesian Skyline Plot	2 runs; 4×10^{7} generations; 2×10^{3} sampling frequency; 25% burn-in
			TRN	ND2-1st	156			

BSP analysis	BEAST	All sequences in *P. vlangalii* Clade IV	HKY+I+G	16S, ND4-1st	700	Strict clock	Coalescent: Bayesian Skyline Plot	2 runs; 4×10^7 generations; 2×10^3 sampling frequency; 25% burn-in
			HKY+I	ND2-1st, ND4-3rd	376			
			HKY	tRNA (ND2+ND4), ND2-2nd, ND2-3rd, ND4-2nd	780			
BSP analysis	BEAST	All sequences in *P. vlangalii* Clade V	HKY	16S, tRNA (ND2+ND4), ND2-1st, ND2-2nd, ND4-2nd	1260	Strict clock	Coalescent: Bayesian Skyline Plot	2 runs; 4×10^7 generations; 2×10^3 sampling frequency; 25% burn-in
			TRN	ND2-3rd, ND4-1st, ND4-3rd,	596			
BSP analysis	BEAST	All sequences in *P. vlangalii* Clade VI	TRN+I+G	16S, ND4-1st	700	Strict clock	Coalescent: Bayesian Skyline Plot	2 runs; 4×10^7 generations; 2×10^3 sampling frequency; 25% burn-in
			HKY	tRNA (ND2+ND4), ND2-1st, ND2-3rd, ND4-2nd, ND4-3rd	1000			
			TRN	ND2-2nd	156			

Table S6. Morphological characters examined in this study.

**metric traits**	
Snout-vent Length	SVL
Tail Length	TL
Head Length	HL
Head Width	HW
Head Height	HH
Internasal space	IS
Distance between nostril and eye	DNE
Fore-limb Length	FLL
Hind-limb Length	HLL
Distance between axilla and groin	DAG
Distance between two axillas	DTA
Fourth finger length	4th FL
Fourth finger claw length	4th FCL
Fourth toe length	4th TL
Fourth toe claw length	4th TCL
**meristic characters**	
Supralabials	SL
Infralabials	IL
Internasals	IN
Nasal	NA
Superciliaris	SC
Upper ciliaris	UC
Lower ciliaris	LC
Scales between lower ciliaris and supralabials	SLS
Scales between mental and gualr fold	SMG
Dorsals	DO
Ventrals	VE
Scales around mid-body	SAM
Fourth toe infratarsals	4th TI

Table S7. Occurrence localities used in ENM.

Location	Longitude	Latitude	Reference
1	36.18	81.56	This study
2	36.22	81.68	This study
3	36.31	81.78	This study
4	36.44	81.97	This study
5	36.67	83.18	This study
6	37.11	84.53	This study
7	37.33	85.47	This study
8	37.43	85.44	This study
9	37.96	91.25	This study
10	38.43	90.12	This study
11	38.9	93.9	This study
12	38.92	90.16	This study
13	39.34	95.32	This study
14	39.43	94.26	This study
15	39.44	94.97	This study
16	36.12	100.44	[12]
17	33.74	102.5	[12]
18	33.94	102.09	[12]
19	34.9	98.21	[12]
20	35.75	94.32	[12]
21	35.88	94.52	[12]
22	36.02	100.26	[12]
23	36.02	97.76	[12]
24	36.37	95.03	[12]
25	36.42	98.13	[12]
26	36.52	96.19	[12]
27	36.65	99.38	[12]
28	36.89	98.53	[12]
29	37.34	97.18	[12]
30	37.89	95.29	[12]
31	37.97	94.41	[12]
32	38.23	90.84	[12]
33	39.42	94.28	[12]
34	33.18	102.6	[28]

35	33.86	102.55	[28]
36	33.95	102.08	[28]
37	34.75	98.11	[28]
38	36.01	97.88	[28]
39	36.17	98.04	[28]
40	36.23	98.11	[28]
41	36.3	98.08	[28]
42	36.38	96.45	[28]
43	36.4	95.07	[28]
44	36.48	99.05	[28]
45	36.55	98.28	[28]
46	36.8	99.08	[28]
47	36.92	93.16	[28]
48	36.93	98.47	[28]
49	37.13	97.23	[28]
50	37.22	97.4	[28]
51	37.85	95.42	[28]
52	38.35	90.15	[28]
53	38.74	93.36	[28]
54	39.44	95.11	[28]
55	34.75	98.12	[90]
56	37.36	97.42	[90]
57	39.3	94.25	[91]
58	39.32	94.3	[91]
59	39.4	94.23	[91]
60	34.62	98.12	[92]
61	37.15	97.58	[92]
62	33.72	102.48	[93]
63	33.18	102.63	[94]
64	33.71	102.49	[94]
65	33.73	102.47	[94]
66	33.75	102.5	[94]
67	33.78	102.55	[94]
68	33.9	102.13	[94]
69	33.95	102.09	[94]

Table S8. Eight bioclimatic factors used in ecological niche modeling.

Bioclimatic variables	Description	Percent contribution	Permutation importance
Bio1	Annul Mean Temperature	48	17.6
Bio2	Mean Diurnal Range (Mean of monthly max -min)	24.3	29.9
Bio3	Isothermality (Bio2/Bio7)	6.2	20.1
Bio7	Temperature Annual Range (Bio5-Bio6)	7.1	25.2
Bio12	Annual Precipitation	3.3	1.6
Bio14	Precipitation of Driest Quarter	2.7	1.6
Bio15	Precipitation of Seasonality (Coefficient of Variation)	4.2	3.3
Bio19	Precipitation of Coldest Quarter	4.1	0.8

Table S9. Morphological data for each clade.

	**Clade II *P. v. vlangalii***					
	Male n=8		Female n=15		Total n=23	
	Range (mm/pieces)	AVG±SD	Range (mm/pieces)	AVG±SD	Range (mm/pieces)	AVG±SD
SVL	47.96~65.29	56.05±6.30	45.33~73.71	58.4±9.32	45.33~73.71	57.5±8.21
TL	44.82~65.15	54.56±6.47	39.17~62.53	50.5±7.37	39.17~65.15	52.0±7.16
HL	14.24~19.18	16.71±1.87	4~19.84	15.8±4.11	4~19.84	16.1±3.40
HW	10.17~13.34	11.76±1.10	10.03~13.93	11.9±1.18	10.03~13.93	11.8±1.13
HH	6.89~9.81	8.61±1.00	6.69~9.73	8.24±0.89	6.69~9.81	8.38±0.93
IS	3.01~4.18	3.43±0.39	2.95~4.34	3.47±0.46	2.95~4.34	3.46±0.42
DNE	2.54~3.7	2.96±0.38	2.24~3.77	2.82±0.36	2.24~3.77	2.87±0.36
FLL	11.12~15.8	13.67±1.53	10.27~16.76	13.7±1.66	10.27~16.76	13.6±1.57
HLL	18.83~23.46	22.09±1.55	15.95~24.36	21.5±2.48	15.95~24.36	21.7±2.15
DAG	22.66~30.57	26.90±2.81	22.68~37.3	29.2±5.10	22.66~37.3	28.3±4.44
DTA	11.16~13.81	12.76±0.88	10.11~16.88	13.5±2.06	10.11~16.88	13.2±1.72
4th FL	4.56~5.92	5.28±0.39	4.24~5.8	5.14±0.51	4.24~5.92	5.19±0.46
4th FCL	2.06~3	2.37±0.30	1.41~3.88	2.56±0.55	1.41~3.88	2.49±0.47
4th TL	7.3~8.95	8.02±0.62	6.17~8.94	7.62±0.74	6.17~8.95	7.77±0.71
4th TCL	1.72~2.62	2.21±0.25	1.39~2.83	2.26±0.39	1.39~2.83	2.24±0.33
SL	13~16	14.00±1.07	12~16	14.53±1.13	12~16	14.35±1.11
IL	14~17	15.50±1.07	13~17	15.07±1.03	13~17	15.22±1.04
IN	4~6	4.75±0.71	3~5	4.53±0.64	3~6	4.61±0.66
NA	3~4	3.63±0.52	3~4	3.93±0.26	3~4	3.83±0.39
SC	9~11	10.00±0.76	8~11	9.53±0.83	8~11	9.70±0.82
UC	10~11	10.13±0.35	8~10	9.53±0.74	8~11	9.74±0.69
LC	8~12	10.00±1.20	9~10	9.53±0.52	8~12	9.70±0.82
SLS	3~4	3.88±0.35	3~4	3.87±0.35	3~4	3.87±0.34
SMG	46~58	50.25±3.92	45~54	50.07±2.55	45~58	50.13±3.00
DO	95~118	105.50±7.37	93~125	107.67±9.73	93~125	106.91±8.87
VE	66~95	80.25±10.74	71~99	84.07±8.43	66~99	82.74±9.24
SAM	102~133	115.00±9.29	103~134	117.60±8.89	102~134	116.70±8.91
4th TI	16~21	18.50±1.77	17~21	18.93±1.33	16~21	18.78±1.48

	Clade III *P. v. nanschanica*					
	Male n=5		Female n=8		Total n=13	
	Range (mm/pieces)	AVG±SD	Range (mm/pieces)	AVG±SD	Range (mm/pieces)	AVG±SD
SVL	50.07~59.28	55.0±4.41	45.91~56.57	51.82±4.12	45.91~56.57	52.9±4.31
TL	48.22~59.17	54.2±4.54	43.69~53.05	48.41±3.64	43.69~53.05	50.3±4.72
HL	14.77~16.6	15.8±0.89	13.62~15.27	14.49±0.71	13.62~15.27	14.9±1.00
HW	11.33~13.6	12.6±1.01	10.48~12.83	11.74±0.79	10.48~12.83	12.0±0.93
HH	7.96~9.04	8.48±0.44	7.19~8.64	7.69±0.56	7.24~8.64	7.96±0.64
IS	3.37~3.85	3.56±0.21	3.15~3.83	3.50±0.22	3.15~3.83	3.52±0.21
DNE	2.31~3.41	2.93±0.46	2.39~2.89	2.65±0.20	2.39~2.89	2.74±0.32
FLL	12.34~14.07	13.5±0.82	12.44~14.21	13.18±0.55	12.44~14.21	13.3±0.64
HLL	21.64~23.54	22.9±0.86	20.19~23.78	21.81±1.28	20.2~23.78	22.1±1.24
DAG	25.92~31.89	28.7±2.69	21.44~31.73	27.26±3.74	21.44~31.73	27.7±3.37
DTA	11.04~13.32	12.4±1.05	10.48~12.65	11.45±0.90	10.65~12.65	11.7±1.03
4th FL	4.76~5.47	5.07±0.30	4.42~5.58	5.02±0.39	4.42~5.39	5.04±0.35
4th FCL	1.76~2.32	2.11±0.24	1.14~2.41	1.97±0.38	1.14~2.19	2.02±0.34
4th TL	7.28~7.56	7.42±0.14	6.66~8.33	7.17±0.51	7.05~8.33	7.25±0.43
4th TCL	1.76~2.35	1.99±0.28	1.49~2.11	1.81±0.24	1.49~1.93	1.87±0.25
SL	14~17	15.40±1.14	14~16	14.63±0.74	14~17	14.92±0.95
IL	14~16	15.00±1.00	14~17	15.13±0.99	14~17	15.08±0.95
IN	5~6	5.20±0.45	4~5	4.50±0.53	4~6	4.77±0.60
NA	3~4	3.20±0.45	3~4	3.88±0.35	3~4	3.62±0.51
SC	9~12	10.00±1.41	8~11	10.00±1.07	8~12	10.00±1.15
UC	9~11	10.40±0.89	9~12	10.38±1.30	9~12	10.38±1.12
LC	8~11	9.60±1.14	9~10	9.50±0.53	8~11	9.54±0.78
SLS	3~4	3.80±0.45	3~4	3.88±0.35	3~4	3.85±0.38
SMG	41~51	44.20±4.09	41~48	43.63±2.33	41~51	43.85±2.97
DO	101~111	107.80±3.96	100~117	108.50±5.88	100~117	108.23±5.05
VE	74~84	79.40±5.08	76~84	80.25±3.20	74~84	79.92±3.84
SAM	114~127	118.60±5.37	112~133	122.13±5.94	112~133	120.77±5.78
4th TI	18~19	18.60±0.55	18~20	19.13±0.83	18~20	18.92±0.76

	Clade IV					
	Male n=14		Female n=17		Total n=31	
	Range (mm/pieces)	AVG±SD	Range (mm/pieces)	AVG±SD	Range (mm/pieces)	AVG±SD
SVL	45.94~53.08	50.07±2.79	46.57~55.92	50.81±2.77	45.21~55.92	50.40±2.75
TL	50.62~64.7	56.16±5.43	46.57~57.83	52.66±3.02	46.57~64.7	54.10±4.58
HL	13.04~14.6	14.48±1.17	12.87~15.81	14.32±0.74	12.49~15.92	14.30±0.95
HW	10.65~11.7	11.40±0.77	10.23~12.83	11.21±0.73	10.23~13.2	11.30±0.74
HH	7.27~7.75	7.44±0.55	6.38~8.26	7.28±0.45	6.38~8.56	7.35±0.49
IS	3.12~3.56	3.27±0.23	3.02~3.51	3.22±0.15	2.93~3.72	3.24±0.19
DNE	2.56~2.86	2.62±0.25	2.27~2.96	2.69±0.23	2.08~2.96	2.66±0.24
FLL	13.6~16.4	14.25±1.01	11.41~14.89	13.75±0.83	11.41~16.4	13.9±0.94
HLL	20.19~22.04	21.59±1.10	19.73~22.12	20.85±0.83	19.73~22.99	21.1±1.02
DAG	23.46~27.91	25.92±1.82	24.92~31.2	26.77±1.77	23.46~31.2	26.3±1.81
DTA	10.51~12.29	11.17±1.06	10.13~12.31	11.02±0.61	9.24~13.03	11.0±0.83
4th FL	4.51~5.5	4.92±0.35	4.23~5.54	4.81±0.42	4.23~5.54	4.86±0.39
4th FCL	2.07~2.36	2.19±0.19	1.73~2.38	2.08±0.20	1.73~2.53	2.13±0.20
4th TL	7.15~7.67	7.42±0.34	6.57~7.69	7.15±0.33	6.57~7.9	7.28±0.36
4th TCL	2.06~2.41	2.16±0.17	1.85~2.29	2.03±0.11	1.85~2.52	2.09±0.16
SL	13~15	14.50±0.65	12~16	13.76±0.97	12~16	14.10±0.91
IL	14~17	15.50±0.85	13~17	15.24±1.03	13~17	15.35±0.95
IN	3~5	4.43±0.65	3~5	4.41±0.71	3~5	4.42±0.67
NA	3~5	4.07±0.62	3~5	3.82±0.53	3~5	3.94±0.57
SC	9~11	10.14±0.66	8~12	10.53±0.94	8~12	10.35±0.84
UC	9~12	10.64±0.93	10~14	10.82±1.07	9~14	10.74±1.00
LC	9~11	10.07±0.73	8~11	10.06±0.90	8~11	10.06±0.81
SLS	4	4	4	4	4	4
SMG	45~59	52.21±3.64	46~58	51.00±3.77	45~59	51.55±3.70
DO	94~118	104.57±6.33	85~112	99.24±7.21	85~118	101.65±7.24
VE	66~89	82.07±5.62	72~89	80.65±5.79	66~89	81.29±5.66
SAM	98~119	109.00±6.61	93~121	106.24±7.22	93~121	107.48±6.98
4th TI	18~22	19.93±1.38	18~22	19.75±1.44	18~22	19.83±1.39

Clade VI *P. v. lidskii*						
	Male n=4		Female n=11		Total n=15	
	Range (mm/pieces)	AVG±SD	Range (mm/pieces)	AVG±SD	Range (mm/pieces)	AVG±SD
SVL	46.22~51.57	48.32±2.85	45.63~50.94	47.68±4.09	45.63~51.64	47.81±3.77
TL	47.06~53.09	50.34±3.05	39.21~54.45	47.07±4.72	39.21~54.45	47.77±4.53
HL	14.11~14.95	14.53±0.42	13.38~14.65	13.43±0.97	11.37~14.95	13.66±0.98
HW	9.76~11.45	11.09±0.51	10.06~11.66	10.68±0.69	9.11~11.66	10.77±0.66
HH	6.66~8.98	7.65±1.20	7.32~8.1	7.49±0.61	6.24~8.98	7.52±0.71
IS	3.14~3.27	3.21±0.06	3.1~3.42	3.21±0.24	2.67~3.58	3.21±0.21
DNE	2.43~2.87	2.72±0.25	2.04~2.54	2.32±0.23	2.03~2.87	2.40±0.28
FLL	10.2~12.96	12.40±0.77	11.43~13.57	12.74±1.02	10.2~14.1	12.66±0.95
HLL	15.41~19.39	18.42±1.46	16.14~20.15	18.21±1.41	15.41~20.15	18.26±1.37
DAG	20.16~24.76	23.63±1.80	23.01~25.6	25.32±3.08	18.48~29.93	24.96±2.88
DTA	9.44~11.11	10.54±0.96	10.09~11.74	10.64±0.78	9.25~11.74	10.62±0.78
4th FL	4.48~4.93	4.74±0.23	4.48~4.87	4.59±0.22	4.25~4.94	4.63±0.23
4th FCL	1.57~2.05	1.91±0.19	1.83~2.3	1.91±0.22	1.57~2.3	1.91±0.21
4th TL	6.11~7.31	6.94±0.39	6.6~7.16	6.71±0.32	6.03~7.31	6.76±0.34
4th TCL	1.52~1.99	1.87±0.13	1.76~2.18	1.93±0.17	1.52~2.18	1.92±0.16
SL	12~13	12.75±0.50	13~15	13.64±0.67	12~15	13.40±0.74
IL	13~15	13.75±0.96	13~15	14.00±0.45	13~15	13.93±0.59
IN	4~5	4.50±0.58	4~5	4.45±0.52	4~5	4.47±0.52
NA	3~4	3.25±0.50	4~4	4.00±0.00	3~4	3.80±0.41
SC	8~10	9±0.82	9~11	9.91±0.54	8~11	9.67±0.72
UC	9~11	10±0.82	9~11	10.09±0.70	9~11	10.07±0.70
LC	9~10	9.75±0.50	9~11	9.73±0.90	9~11	9.73±0.80
SLS	4	4	3~4	3.91±0.30	3~4	3.93±0.26
SMG	46~49	47.25±1.50	40~51	45.09±3.27	40~51	45.67±3.02
DO	87~98	91.50±5.45	86~104	91.82±6.00	86~104	91.73±5.66
VE	67~81	74.50±7.05	64~79	73.27±4.92	64~81	73.60±5.32
SAM	92~107	101.25±6.90	86~112	96.09±7.31	86~112	97.47±7.35
4th TI	16~18	17.25±0.96	15~19	17.45±1.13	15~19	17.40±1.06

Table S10. Loading and cumulative variation for the principal components.

metric traits	Male			Commonality	Female			Commonality
	PC1	PC2	PC3		PC1	PC2	PC3	
SVL	**0.939**	-0.232	-0.006	0.935	**0.955**	0.074	-0.128	0.934
TL	**0.746**	**0.434**	0.372	0.883	**0.671**	-0.329	0.005	0.559
HL	**0.779**	**-0.460**	-0.074	0.824	**0.636**	**0.468**	-0.184	0.658
HW	**0.762**	-0.132	**0.450**	0.800	**0.898**	-0.110	-0.210	0.862
HH	**0.746**	**-0.524**	0.032	0.833	**0.822**	0.070	-0.259	0.748
IS	**0.744**	-0.220	0.380	0.747	**0.815**	-0.048	-0.214	0.712
DNE	**0.653**	-0.270	0.055	0.502	**0.722**	0.049	-0.045	0.526
FLL	**0.606**	**0.696**	0.242	0.911	**0.779**	-0.252	0.167	0.698
HLL	**0.760**	**0.409**	0.152	0.769	**0.833**	-0.330	-0.002	0.803
DAG	**0.860**	-0.009	0.111	0.752	**0.897**	0.015	-0.213	0.850
DTA	**0.869**	-0.221	-0.127	0.819	**0.897**	0.112	-0.156	0.842
4th FL	**0.739**	-0.025	**-0.475**	0.773	**0.714**	-0.135	0.390	0.680
4th FCL	**0.733**	0.324	-0.277	0.719	**0.777**	0.339	0.374	0.858
4th TL	**0.815**	0.066	-0.392	0.822	**0.730**	-0.320	0.313	0.733
4th TCL	**0.657**	**0.424**	**-0.415**	0.784	**0.680**	**0.472**	0.366	0.820
Variance explanation rate %	58.543	12.384	8.217		63.018	6.655	5.540	

meristic characters	PC1	PC2	PC3	PC4	Commonality
SL	**0.633**	-0.190	0.371	-0.252	0.638
IL	**0.711**	0.133	-0.082	-0.155	0.555
IN	0.191	-0.198	**0.481**	**0.632**	0.707
NA	0.228	**0.469**	-0.185	0.200	0.346
SC	0.293	**0.566**	**0.451**	-0.079	0.616
UC	0.069	**0.627**	**0.431**	0.219	0.631
LC	-0.005	**0.503**	0.224	**-0.556**	0.613
SLS	0.256	0.339	-0.341	0.079	0.303
SMG	**0.482**	**0.573**	-0.324	0.111	0.678
DO	**0.793**	-0.376	0.115	0.001	0.784
VE	**0.737**	-0.135	-0.101	0.214	0.617
SAM	**0.654**	**-0.429**	0.037	-0.281	0.692
4th TI	**0.652**	0.099	-0.284	0.093	0.524
Variance explanation rate %	26.223	15.967	9.048	8.026	

Note: Bold indicates that the load value is greater than 0.4.

References

12 Chen, Y.J.; Zhu, L.; Wu, Q.N.; Hu, C.C.; Qu, Y.F.; Ji, X. Geological and climatic influences on population differentiation of the *Phrynocephalus vlangalii* species complex (Sauria: Agamidae) in the northern Qinghai-Tibet Plateau. *Mol. Phylogenet. Evol.* **2022**, *169*, 107394; DOI: 10.1016/j.ympev.2022.107394.

13 Jin, Y.T.; Brown, R.P. Species history and divergence times of viviparous and oviparous Chinese toad-headed sand lizards (*Phrynocephalus*) on the Qinghai-Tibetan Plateau. *Mol. Phylogenet. Evol.* **2013**, *68*, 259–68; DOI: 10.1016/j.ympev.2013.03.022.

14 Jin, Y.T.; Yang, Z.S.; Brown, R.P.; Liao, P.H.; Liu, N.F. Intraspecific lineages of the lizard *Phrynocephalus putjatai* from the Qinghai-Tibet Plateau: impact of physical events on divergence and discordance between morphology and molecular markers. *Mol. Phylogenet. Evol.* **2014**, *71*, 288–297; DOI: 10.1016/j.ympev.2013.11.004.

27 Jin, Y.T.; Brown, R.P.; Liu, N.F Cladogenesis and phylogeography of the lizard *Phrynocephalus vlangalii* (Agamidae) on the Tibetan plateau. *Mol. Ecol.* **2008**, *17*, 1971–82; DOI: 10.1111/j.1365-294X.2008.03721.x.

28 Jin, Y.T.; Liu, N.F. Phylogeography of *Phrynocephalus erythrurus* from the Qiangtang Plateau of the Tibet Plateau. *Mol. Phylogenet. Evol.* **2010**, *54*, 933–940; DOI: 10.1016/j.ympev.2009.11.003.

74 Rassmann, K. Evolutionary age of the Galápagos iguanas predates the age of the present Galápagos Islands. *Mol. Phylogenet. Evol.* **1997**, *7*, 158–172; DOI: 10.1006/mpev.1996.0386.

75 Arèvalo, E.; Davis, S.K.; Sites, J.W. Mitochondrial DNA sequence divergence and phylogenetic relationships among eight chromosome races of the *Sceloporus grammicus* complex (Phrynosomatidae) in central Mexico. *Syst. Biol.* **1994**, *43*: 387-418; DOI: 10.1093/sysbio/43.3.387.

76 Macey, J.R.; Schulte, J.A.; Ananjeva, N.B.; Larson, A. Phylogenetic relationships among agamid lizards of the *Laudakia caucasia* species group: testing hypotheses of biogeographic fragmentation and an area cladogram for the Iranian Plateau. *Mol. Phylogenet. Evol.* **1998**, *10*, 118–131; DOI: 10.1006/mpev.1997.0478.

77 Tong, H.; Jin, Y. The complete mitochondrial genome of an agama, *Phrynocephalus putjatia* (Reptilia, Squamata, Agamidae). *Mitochondrial DNA A* **2014**, *27*, 1028–1029; DOI: 10.3109/19401736.2014.926538.

78 Zhu, L.; Liao, P.; Tong, H.; Jin, Y. The complete mitochondrial genome of the subspecies, *Phrynocephalus erythrurus parva* (Reptilia, Squamata, Agamidae), a toad-headed lizard dwell at highest elevations of any reptile in the world. *Mitochondrial DNA A* **2014**, *27*, 703–704; DOI: 10.3109/19401736.2014.913151.

79 Jin, Y.T.; Brown, R.P. Partition number, rate priors and unreliable divergence times in Bayesian phylogenetic dating. *Cladistics* **2018**, *34*, 568–573; DOI: 10.1111/cla.12223.

80 Li, J.; Guo, X.; Chen, D.; Wang, Y. The complete mitochondrial genome of the Yarkand toad-headed agama, *Phrynocephalus axillaris* (Reptilia, Squamata, Agamidae). *Mitochondrial DNA* **2013**, *24*, 234–236; DOI: 10.3109/19401736.2012.752477.

81 Chen, D.; Zhou, T.; Guo, X. The complete mitochondrial genome of *Phrynocephalus forsythii* (Reptilia, Squamata, Agamidae), a toad-headed agama endemic to the Taklamakan Desert. *Mitochondrial DNA A* **2016**, *27*, 4046–4048; DOI: 10.3109/19401736.2014.1003837.

82 Fu, C.; Chen, W.; Jin, Y. The complete mitochondrial genome of *Phrynocephalus guinanensis* (Reptilia, Squamata, Agamidae). *Mitochondrial DNA A* **2016**, *27*, 1103–1104; DOI: 10.3109/19401736.2014.933320.

83 Li, D.; Song, S.; Chen, T.; Zhang, C.; Chang, C. Complete mitochondrial genome of the desert toad-headed agama, *Phrynocephalus przewalskii* (Reptilia, Squamata, Agamidae), a novel gene organization in vertebrate mtDNA. *Mitochondrial DNA* **2015**, *26*, 696–697; DOI: 10.3109/19401736.2013.843079.

84 Li, D.; Guo, J.; Zhou, X.; Chang, C.; Zhang, S. The complete mitochondrial genome of *Phrynocephalus helioscopus* (Reptilia, Squamata, Agamidae). *Mitochondrial DNA A* **2016**, *27*, 1846–1847; DOI: 10.3109/19401736.2014.971253.

85 Chen, D.; Guo, X.; Li, J. The complete mitochondrial genome of secret toad-headed agama, *Phrynocephalus mystaceus* (Reptilia, Squamata, Agamidae). *Mitochondrial DNA* **2013**, *25*, 19–20; DOI: 10.3109/19401736.2013.775269.

86 Song, S.; Li, D.; Zhang, C.; Jiang, K.; Zhang, D.; Chang, C. The complete mitochondrial genome of the color changeable toad-headed agama, *Phrynocephalus versicolor* (Reptilia, Squamata, Agamidae). *Mitochondrial DNA A* **2016**, *27*, 1121–1122; DOI: 10.3109/19401736.2014.933329.

87 Shuang, L.; Liu, L.; Song, S. The complete mitochondrial genome of Grumgzimailo's toad-headed agama, *Phrynocephalus grumgrizimailoi* (Reptilia, Squamata, Agamidae). *Mitochondrial DNA A* **2014**, *27*, 1581–1582; DOI: 10.3109/19401736.2014.958678.
88 Shao, M.; Ma, L.; Zhang, G.; Wang, Z. The complete mitochondrial genome of the toad-headed lizard, *Phrynocephalus albolineatus* (Reptilia, Squamata, Agamidae). *Mitochondrial DNA A* **2017**, *28*, 137–138; DOI: 10.3109/19401736.2015.1111359.
89 Peng, L.; Huang, S. GenBank. Available online: https://www.ncbi.nlm.nih.gov/nuccore/MK411595 (accessed on 25 August 2023).
90 Li, J.Q. Life history evolution of *Phrynocephalus* (Agamidae) along altitudinal gradients on Tibetan Plateau. Doctoral Dissertation, Lanzhou University, Lanzhou, China, 2013. (In Chinese with English abstract)
91 Yu, W. Altitudinal differences in reproductive life history traits and offspring growth of *Phrynocephalus vlangalii*. Master Dissertation, Northeast Forestry University, Harbin, China, 2021. (In Chinese with English abstract)
92 Niu, C.K. Study on the personality and gut microbiota of *Phrynocephalus vlangalii*. Master Dissertation, Lanzhou University, Lanzhou, China, 2022. (In Chinese with English abstract)
93 Wan, H.; Qi, Y; Guo, X.; Wang, Y. Preliminary study on sexual dimorphism and seasonal variation of the belly patch and tail-tip badge of the adult toad-headed lizard *Phrynocephalus vangalii hongyuanensis*. *Sichuan J. Zool.* **2011**, *30*, 54–58.
94 Liu, L.; Guo, X.; Wang, Y. Genetic variation and diversity of *Phrynocephalus vlangalii hongyuanensis* in Zoige wetland inferred from ND4-tRNAleu Gene. *Zool. Res.* **2008**, *29*, 121–126. (In Chinese with English abstract)
